# A bibliometric analysis of statistical terms used in American Physical Therapy Association journals (2011-2012): evidence for educating physical therapists

**DOI:** 10.1186/s12909-016-0641-1

**Published:** 2016-04-22

**Authors:** Julie K. Tilson, Katie Marshall, Jodi J. Tam, Linda Fetters

**Affiliations:** Division of Biokinesiology and Physical Therapy, University of Southern California, 1540 Alcazar St, CHP 155, Los Angeles, CA 90089 USA; The Center for Physical Therapy, Long Beach, CA USA; Physiotherapy Associates, San Diego, CA USA

**Keywords:** Statistics, Evidence based practice, Physical therapy, Bibliographic analysis, Education, Curriculum design

## Abstract

**Background:**

A primary barrier to the implementation of evidence based practice (EBP) in physical therapy is therapists’ limited ability to understand and interpret statistics. Physical therapists demonstrate limited skills and report low self-efficacy for interpreting results of statistical procedures. While standards for physical therapist education include statistics, little empirical evidence is available to inform what should constitute such curricula. The purpose of this study was to conduct a census of the statistical terms and study designs used in physical therapy literature and to use the results to make recommendations for curricular development in physical therapist education.

**Methods:**

We conducted a bibliometric analysis of 14 peer-reviewed journals associated with the American Physical Therapy Association over 12 months (Oct 2011-Sept 2012). Trained raters recorded every statistical term appearing in identified systematic reviews, primary research reports, and case series and case reports. Investigator-reported study design was also recorded. Terms representing the same statistical test or concept were combined into a single, representative term. Cumulative percentage was used to identify the most common representative statistical terms. Common representative terms were organized into eight categories to inform curricular design.

**Results:**

Of 485 articles reviewed, 391 met the inclusion criteria. These 391 articles used 532 different terms which were combined into 321 representative terms; 13.1 (sd = 8.0) terms per article. Eighty-one representative terms constituted 90 % of all representative term occurrences. Of the remaining 240 representative terms, 105 (44 %) were used in only one article. The most common study design was prospective cohort (32.5 %).

**Conclusions:**

Physical therapy literature contains a large number of statistical terms and concepts for readers to navigate. However, in the year sampled, 81 representative terms accounted for 90 % of all occurrences. These “common representative terms” can be used to inform curricula to promote physical therapists’ skills, competency, and confidence in interpreting statistics in their professional literature. We make specific recommendations for curriculum development informed by our findings.

**Electronic supplementary material:**

The online version of this article (doi:10.1186/s12909-016-0641-1) contains supplementary material, which is available to authorized users.

## Background

Physical therapist education that meets international standards [1] includes “applied statistics”. In the United States, the licensure exam for physical therapists includes statistics, described as “Statistics (e.g., t‐test, chi‐square, correlation coefficient, ANOVA, likelihood ratio)” [2]. Yet, a primary barrier to the implementation of evidence based practice (EBP) in physical therapy is therapists’ inability to understand and interpret statistics [3–5]. Physical therapists report low self-efficacy for interpreting results of statistical procedures [4, 5] and demonstrate corresponding limited skills with objective testing [5, 6]. For example, after an intensive 6-month educational program to improve EBP skills, experienced physical therapists demonstrated poor skills for interpreting statistical results and reported persistent frustration about difficulty understanding statistical methods and interpreting results from research literature [5]. Those physical therapists also reported a need for greater learning opportunities in the area of statistics. Few resources are available to facilitate evidence-based development of curricula to promote physical therapist practitioners’ statistical knowledge for understanding and using evidence to inform practice.

While credentialing standards suggest that statistical concepts are part of physical therapist education, this has not resulted in sufficient therapist confidence and skills for using statistical concepts as evidence based practitioners. Myriad physical therapy-specific textbooks are available on the topics of ‘statistics in rehabilitation’ and ‘evidence based practice’ and a recent educational guideline for teaching evidence based practice [7, 8] recommends that educational programs include a number of categories of statistics (descriptive, inferential, clinically meaningful). However, scant empirical evidence is available regarding the statistical terms typically reported in physical therapy literature. Understanding the characteristics of statistical terminology reported in physical therapy literature would assist faculty in determining educational content that would best support practitioners’ use of research evidence for practice.

Bibliometric analyses have been used to describe the statistical procedures used in discipline-specific literature [medicine [9], rehabilitation [10], surgery [11], pharmacy [12], ophthalmology [13]] and in specific journals [*Journal of Foot and Ankle Surgery* [14], *Burns* [15], *Pediatrics* [16], *New England Journal of Medicine*] [17]. While the usefulness of these reports for supporting educational curricula has been identified [10, 14], the design and intent of such studies have not typically been aimed at informing educational design for clinician consumers of research.

In the past 15 years, two studies have described statistical methods used in physical therapy literature [18, 19]. Bandy reviewed 2 years of research reports in *Physical Therapy* (2000–2002) and reported the 25 most commonly used categories of statistical methods [18]. Roush and colleagues [19] report the most common statistical methods used in a two year period (2009–2010) in 15 of 16 journals defined by Fell et al. [20] as core physical therapy journals (those most frequently cited by the flagship journals of four national physical therapy associations: United States, Canada, United Kingdom, and Australia).

While these studies provide a starting point for understanding what physical therapists encounter in professional literature, gaps in the research remain. First, both studies address only the statistical methods used in the studies reviewed. Hence, statistical terms beyond those used to describe a statistical ‘method’ used for analysis are not identified (e.g., terms associated with statistical design and interpretation of results). Second, both studies present categories of statistical methods (e.g., “epidemiology”, “nonparametric”) without discriminating the relative frequency of terms within the categories. Third, in the more recent study [19], only 6.0 % of articles reviewed were from physical therapy-specific journals. Therefore, many of the articles in the data set are unlikely to represent the types of articles that physical therapists would read most often to inform their clinical decision-making. Fourth, several specialty-practice areas of physical therapy are likely under-represented (e.g., pediatrics, women’s health, acute care) based on the journals included in the two previous analyses. Fifth, neither study provides information about the frequency of various study designs in their cohort of articles. Because study design is integrally linked to statistical methods [21], concurrent information about study design and statistical terms would be valuable for teaching and learning.

This study takes a unique approach to addressing the question, “What are the most common statistical terms and research concepts physical therapists are likely to encounter in the physical therapy literature that need to be included in professional education curricula?” By identifying the most common statistical *terms* rather than statistical *methods or categories of methods*, we more explicitly define learning needs. Statistical term frequency better reflects readers’ experience encountering statistical terminology as they navigate research articles. This study provides results exclusive to physical therapy across a spectrum of practice areas. By including all peer-reviewed journals associated with the American Physical Therapy Association and its components, our results are specific to physical therapy literature and include all major physical therapy specialty areas within United States practice patterns. Finally, we analyzed study design frequency, a critical component for informed curricular design, published in American Physical Therapy Association journals.

The purpose of this study was to enumerate the frequency of use of statistical terms and study designs in physical therapy literature. From this enumeration, we make recommendations for the development of educational curricula to promote physical therapists’ self-efficacy and skills for navigating the statistical terms associated with their professional research literature.

## Methods

### Inclusion and exclusion criteria

Contents of every peer-reviewed journal associated with the American Physical Therapy Association (14 journals; Table 1) were reviewed over 12 months (Oct 2011-Sept 2012). The 1-year time frame was chosen based on previous studies and the authors’ estimate of a sufficient sample size. The start date was selected based on published article availability (data collection began in October 2012, the month immediately following the selected time frame). All research and case series and case reports were included. Article types that did not include original data (e.g., perspective papers, clinical commentaries, narrative/literature reviews, clinical imaging reports) were excluded. Items listed in a journal’s table of contents that did not have an abstract were not considered (e.g., editorials, lectures, conference abstracts, organizational announcements/news, letters, book reviews).

**Table 1:** Peer-reviewed journals associated with the American Physical Therapy Association and its components in alphabetical order

1. Cardiopulmonary Physical Therapy Journal
2. International Journal of Sports Physical Therapy
3. Journal of Acute Care Physical Therapy
4. Journal of Geriatric Physical Therapy
5. Journal of Neurologic Physical Therapy
6. Journal of Orthoepaedic & Sports Physical Therapy
7. Journal of Physical Therapy Education
8. Journal of Women’s Health Physical Therapy
9. Orthopaedic Physical Therapy Practice
10. Pediatric Physical Therapy
11. Physical Therapy Journal
12. Physical Therapy Journal of Policy, Administration and Leadership
13. Rehabilitation Oncology
14. Sports Health^a^

^a^Only articles published in the “Sports Physical Therapy” category of this multi-disciplinary journal were considered for inclusion

### Data collection

#### Statistical terms

Two raters (KM, JJT) were trained to collect the data. Intraclass correlation coefficient (ICC; 2-way mixed approach with absolute agreement) was used to determine rater reliability for identifying the correct list of terms for an article. Raters scored 12 articles randomly selected (using random number generator) from the most recent issue of included journals as of September 2012. Raters had excellent reliability, ICC > 0.97, compared with a gold standard (primary author) and with each other.

Each journal’s table of contents was reviewed electronically for journal issues published within the study time period (Oct 2011-Sept 2012). Every article listed in the table of contents was screened for inclusion/exclusion by reviewing its title, abstract, content, and type as designated by the journal (e.g., “research report”, “perspective”). Articles that met the inclusion criteria were downloaded and printed. Raters used a pen to mark every unique statistical term used in the methods, results, figures, tables, and discussion in each article. The discussion section was included because pilot work revealed that authors often use clinically meaningful statistical terms (e.g., minimal clinically important difference and minimal detectible change) exclusively in the discussion section.

Terms were recorded in a Microsoft Excel® file along with reference information for the corresponding article. Terms were recorded once per article, regardless of the number of recurrences within the article. No judgments or inferences were made regarding appropriateness of statistical term use.

#### Study design

Raters recorded the study design as published for each article. Articles that did not designate a study design were assigned one upon review of the abstract by the primary author. Articles containing more than one study design were assigned to the dominant study design based on review of the methods and results.

### Data analysis

#### Statistical terms

Representative terms were identified by combining terms representing the same statistical test or concept into a single, term by authors (JKT, LF) with reference to rehabilitation-specific statistical texts [22, 23]. For example, the terms “statistical significance”, “statistically significant”, “significant”, and “significance” were combined into the representative term “statistical significance”.

For each representative term, total occurrences were determined by summing the number of articles that used that term. Cumulative percentages were calculated starting with the representative term with the highest number of total occurrences. Representative terms representing 90 % cumulative percentage, deemed “common representative terms”, were grouped into categories to facilitate interpretation of the results. Categories were developed iteratively by the authors (JKT, LF). Each common term was assigned to one category.

#### Study design

Variations of the same study design were combined by the primary author. For example, the terms “cross-sectional observational”, “cross-sectional cohort”, “cross-sectional descriptive”, “descriptive cross-sectional cohort”, and “observational cross-sectional” were combined into a single design “cross-sectional”. Cohort studies were designated as either prospective or retrospective cohort studies. The proportion of each study design’s contribution to the data set was calculated.

## Results

**Fig. 1:**
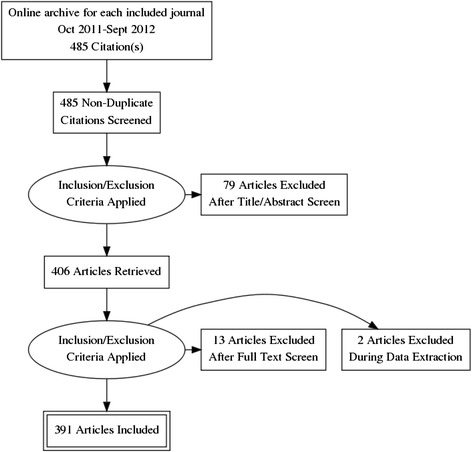
Flowchart of included articles

Among 485 articles reviewed, 391 met the inclusion criteria and were included in the analysis (Fig. 1). Table 2 lists the contribution of each journal to the data set.

**Table 2:** Contribution of each journal to articles included and excluded; ordered by largest number of included articles

Journal	Articles included (%)	Articles excluded (%)
Physical Therapy Journal	114 (29.2)	20 (21.3)
Journal of Orthoepaedic & Sports Physical Therapy	65 (16.6)	27 (28.7)
International Journal of Sports Physical Therapy	44 (11.3)	7 (7.4)
Pediatric Physical Therapy	37 (9.5)	3 (3.2)
Journal of Physical Therapy Education	22 (5.6)	9 (9.6)
Journal of Geriatric Physical Therapy	20 (5.1)	6 (6.4)
Journal of Neurologic Physical Therapy	19 (4.9)	5 (5.3)
Orthopaedic Physical Therapy Practice	18 (4.6)	2 (2.1)
Cardiopulmonary Physical Therapy Journal	14 (3.6)	2 (2.1)
Journal of Women's Health Physical Therapy	10 (2.6)	2 (2.1)
Rehabilitation Oncology	9 (2.3)	0 (0)
Journal of Acute Care Physical Therapy	9 (2.3)	3 (3.2)
Sports Health (“Sports Physical Therapy” articles)	6 (1.5)	8 (8.5)
Physical Therapy Journal of Policy, Administration and Leadership	4 (1.2)	0 (0)
Total	391(100)	94 (100)

### Statistical terms

Initially, 532 terms were identified; those were consolidated to 321 representative terms. Each representative term combines an average of 1.65 (sd = 1.81) of the original 532 terms. All representative terms, with the terms that were combined to create them, are available online [see Additional file 1]. Mean (sd) number of terms per article was 13.1(8.0) with the range: 0–39. One hundred sixty-one articles (41.2 %) used 0–10 terms, 159 articles (40.7 %) used 11–20 terms, 64 articles (16.4 %) used 21–30 terms, and 7 articles (1.8 %) used 31–39 terms.

**Fig. 2:**
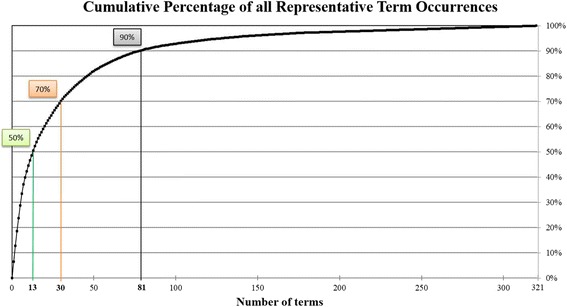
Illustrates the number of terms that account for the cumulative percentage of all representative statistical term occurrences

Fifty percent of all representative term occurrences were accounted for by 13 representative terms, 70 % by 30 terms, and 90 % by 81 “most common” representative terms (Fig. 2). Each of the remaining 240 representative terms was used by eight or fewer articles; 105 representative terms were used in just one article. Eight categories identified by the authors to sub-group the 81 most common representative terms are defined in Table 3. Table 4 lists the most common representative terms by category. Categories are listed in descending order of each category’s most common term.

**Table 3:** Categories for most common representative statistical terms

1. Between Group(s) Comparison	Statistical terms relating to comparisons between groups, usually groups of people
2. Clinically Meaningful Statistics	Statistical expressions that provide the clinician and/or the patient with information that is meaningful to clinical care
3. Describing Variables	Names of categories or types of variables
4. Diagnostic Statistics	Statistical terms that reflect the accuracy and usefulness of tests for the diagnostic process
5. Measures of Association	Statistical tests that determine the similarity of change in variables or the values of two or more variables at one point in time
6. Measures of Central Tendency	Statistical terms that express the most typical values of a measure and the range or distribution of those values
7. Results Terms	Statistical terms that are used to express the result of a statistical test
8. Sundry Statistical Terms	Statistical terms or processes that do not fit well into the other categories.

**Table 4:**
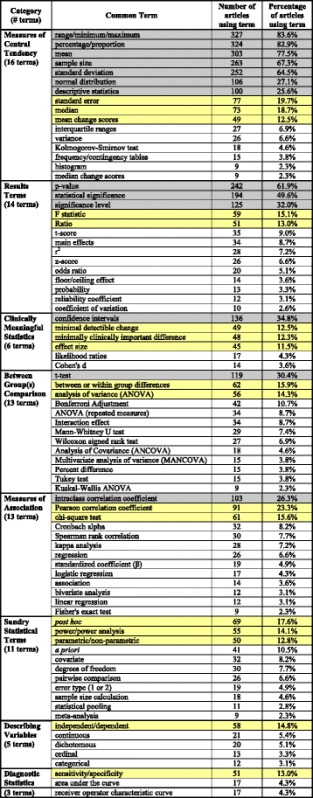
Most common representative statistical terms (representing 90 % of all representative term occurrences) listed by category. Terms highlighted in green represent 50 % of representative term occurrences; green + yellow highlights represent 70 % of representative term occurrences

See Online Appendix 1 to view additional (related) terms associated with some terms in this list

### Study design

Twenty-one study designs were represented within the 391 articles. Table 5 includes the study designs in descending order of frequency. The most common design was prospective cohort studies (32.5 %), followed by case reports (16.9 %), and randomized controlled trials (7.9 %). A list of the most common representative statistical terms for each of the five most common study designs (prospective cohort, case report, randomized controlled trial, cross sectional, and systematic review) is available online [see Additional file 1]. Study design frequency for each journal is available online [see Additional file 2].

**Table 5:** Frequency of study design

Study type	Number of articles (%)
Prospective cohort	127 (32.5)
Case report	66 (16.9)
Randomized controlled trial	31 (7.9)
Cross sectional	29 (7.4)
Systematic review	19 (4.9)
Case series	17 (4.3)
Psychometric analysis	17 (4.3)
Retrospective cohort	17 (4.3)
Survey	13 (3.3)
Qualitative	10 (2.6)
Case control	8 (2.0)
Technological report	6 (1.5)
Secondary analysis	6 (1.5)
Cross over	6 (1.5)
Cadaver or animal	5 (1.3)
Quasi experimental	4 (1.0)
Model analysis	4 (1.0)
Mixed method	2 (0.5)
Bibliometric analysis	2 (0.5)
Policy analysis	1 (0.3)
Economic analysis	1 (0.3)
Total	391 (100)

## Discussion

Readers of physical therapy literature encounter hundreds of statistical terms. We identified 321 representative terms, representing 532 total terms, in 391 articles from one selected year of American Physical Therapy Association-associated peer-reviewed journals. However, 25 % (i.e., 81 common representative terms/321 representative terms) of those terms represent 90 % of the occurrences of representative terms in the sample. Educational curricula should focus, though not exclusively, on the common representative terms. The remaining 75 % (*n* = 240) of representative terms are rarely used, and these terms may represent a challenge to physical therapists, both with respect to skills needed to understand their scientific literature and to their self-efficacy in understanding and using research evidence to inform practice.

There is also diversity in the study designs represented in our sample, however, prospective cohort and case studies represented about half of all studies. Wiles et al. identified a steady increase in higher quality study designs (i.e. systematic reviews and randomized controlled trials) in *Physical Therapy* from 1945 to 2010. Given this trend and the larger impact that these designs should have on clinical decision making, it is logical to emphasize statistical terms related to randomized controlled trials and systematic reviews in curricula, despite their relatively small representation in the sample (7.9 and 4.9 %, respectively). Furthermore, new funding for comparative and cost-effectiveness research in healthcare in the United States [24] and specifically physical therapy [25] may increase the frequency of economic analyses and consequently the need for readers to understand terms associated with those study designs.

The empirical evidence revealed in this bibliographic analysis can inform the statistical and research curricula of physical therapist education programs. Existing physical therapy curricula related to statistics and research methods are likely to overlap with our findings, particularly if they are based upon a combination of previous studies [10, 18, 19], common texts, instructor experience, and deductive reasoning. However, our unique methods have identified new information that facilitates more meaningful and relevant statistics curricula in both professional and post-professional physical therapist education.

This study is the first to our knowledge to analyze the statistical terms used in a body of literature rather than focusing exclusively on the statistical methods reported. Further, by selecting all peer-reviewed journals associated with the American Physical Therapy Association, we specifically examined physical therapy-related literature and were inclusive of the major sub-specialties of practice in the United States.

Our inclusion criteria did not discriminate by journal quality and did not include multi-disciplinary journals (e.g., *Spine, Stroke*) or international physical therapy-specific journals. Rousch and colleagues [19] identified 25 statistical methods or categories of methods in 16 journals, that while not exclusively physical therapy-specific, were international, multi-disciplinary, and high impact. For the statistical methods with individual frequency reported (18 items) in that study, there is excellent overlap with our findings. Thirteen methods accounted for 96.5 % cumulative frequency. All 13 of those methods were captured in our list of common representative terms. However, because we included all statistical terms, our results provide a more comprehensive picture of what physical therapists need to know to understand their professional literature.

To the extent that the lists can be compared, our common representative terms list and the common methods identified by Bandy [18] in *Physical Therapy* from 2000 to 2002 are also similar. The most common statistical methods (11 methods that account for 85 % cumulative frequency) identified by Bandy are accounted for in our common representative terms. Because Bandy assessed frequency of statistical method use (not frequency of statistical terms reported), inferences about changes in statistical term usage during the 10-year gap between studies cannot be drawn reliably.

Based on our findings we recommend that curricula for teaching statistical knowledge to physical therapists should:

1. Focus on the most common representative statistical terms in a clinically relevant context.
2. Use the eight categories provided to organize curricula, recognizing that learning crosses categories.
3. Use study design frequency and anticipated changes in publication patterns to adjust learning priorities.
4. Address skills for independent learning of unfamiliar statistical terms.
5. Provide reference and refresher resources for practicing clinicians.

*Recommendation 1:* Focus on the most common statistical terms in a clinically relevant context.

The most common terms that physical therapists encounter in their professional literature should be a major driver of statistical curricular emphases. Based on evidence for learning EBP skills in general [26], statistical learning is most likely to be effective when it is integrated into the process of solving clinical problems. For example, one of the articles in our data set [27] conducted a randomized controlled trial comparing cervical thrust manipulation to thoracic thrust manipulation for persons with neck pain. A lesson could be developed that asks learners to develop a care plan for a patient with neck pain presenting similarly to those in the study. By exploring the statistical design and results of the study, learners could determine that although the study had sufficient power there was not a statistically significant difference between groups for pain pressure thresholds at any location (e.g., C5-C6: f-value = 1.233, *p*-value = .210). In this example, four terms can be explored in a context that has meaning for a practicing clinician. In this paper alone, 19 terms from the common representative terms list could be built into a clinically relevant learning experience about statistics and statistical terms. Time available in an individual curriculum would dictate how many of the common representative terms are included. Further, educational philosophy and practices beyond the scope of this manuscript should drive how the terms are taught.

*Recommendation 2:* Use the eight categories provided to organize curricula; recognizing that learning crosses categories.

The eight categories provided in this study were generated in response to the results of the study. Hence, they are not the common set of categories found in statistics texts (though they do overlap). We propose that these categories are useful for organizing common statistics in physical therapy education. However, we do not recommend teaching or learning from the categories in isolation. Particularly, terms in the Results Terms category are naturally linked to the statistical tests that generate those results. For example, using the same article as the previous example [27], a lesson could illustrate how terms from several categories are linked in reporting the study result: means (category: Measures of Central Tendency) on the numeric pain rating scale, represent continuous data (category: Describing Variables), and can be used to generate a within group difference (category: Between Group(s) Comparison) with a 95 % confidence interval (category: Clinically Meaningful Statistics).

*Recommendation 3:* Use study design frequency and anticipated changes in publication patterns to adjust learning priorities.

The research design of a study determines the statistical methods appropriate to analyze the findings. Curricula for understanding statistical terms in the literature should be developed with attention to study designs that may become a larger proportion of physical therapy literature in the future; thus, preparing physical therapists to effectively access research evidence over time. For example, with the increasing prevalence of systematic reviews [28], terms such as relative risk, forest plot, and standardized mean difference should be in curricula even though they are not classified as common representative terms in our study.

*Recommendation 4:* Address skills for independent learning of unfamiliar statistical terms.

This study identified what could be a major barrier to physical therapists’ ability to achieve self-efficacy and mastery in understanding physical therapy literature. That is, the vast number of terms – many of which appear so rarely as to have limited justification to include in curricula. Examples of terms with one occurrence in our study include Tucker-Lewis Index, zero-order analysis, stochastic independence, and Pitman test. We expect that uncommon terms reduce both comprehension and confidence among readers. Curricula should include instruction on how readers can learn uncommon terms. Example strategies include both where to look-up unfamiliar terms and how to decide when it is necessary, and to what extent, to determine the meaning of an unfamiliar term.

*Recommendation 5:* Provide reference and refresher resources for practicing clinicians.

Given the extensive number of terms identified in this study, mastery of statistical knowledge for needed interpreting and applying physical therapy-related research literature upon graduation from a physical therapist education program is unrealistic. Further, clinicians may return to the topic after graduation and require review of previously learned material. Development of physical therapy-specific online reference materials and educational opportunities for clinicians may help to break down the ‘statistical knowledge’ barrier between therapists’ desire to use evidence to inform their clinical decision-making and their ability to do so.

### Limitations

We limited our journal selection to those published by the American Physical Therapy Association and its components over one selected year. Thus, our findings are biased toward physical therapists in the U.S. reading literature published by components of their professional association in a specific time period. Further, physical therapists read many more journals than the 14 we selected. We chose not to use previously developed lists of core journals that publish clinical trials of physical therapy interventions [29] or the core physical therapy journals defined by Fell et al. [20] because those journal lists, while useful, are not specific to physical therapy and do not represent a broad spectrum of practice. Thus, physical therapists may encounter a different set of common terms as they read articles in multi-disciplinary journals and as research reporting changes over time. However, the considerable overlap of our results with others [18, 19] suggests that the difference is likely to be small.

The process of combining like terms into representative terms was by its nature, subjective. It is possible that others might have grouped common representative terms differently. We provided the full list of combined terms for transparency and to support replication of the study. Similarly, our process for creating categories to organize the most common representative terms was iterative and there are alternative organizational strategies. Because the statistical terms were not influenced by the categories, terms can be freely reorganized into various categories without diminishing the validity of the results. Finally, the method used to classify each article’s study design has some risk of bias. We used investigator-reported study design and collapsed those into 21 categories, which required interpretation by the authors.

### Research implications

Research is needed into how physical therapist education can address the barrier that ‘inability to understand and interpret statistics’ presents to the implementation of EBP in physical therapy. There is a need to assess the congruence between our findings and what physical therapy education programs are actually teaching. Further, there is a need to establish that knowledge of the common representative terms identified in this study results in improved therapist skills, competency, and confidence in applying research evidence in practice. Finally, replication of this study is needed to assess changes in physical therapy literature over time. Such work might also be expanded to analyze the statistical terms in subsets of literature such as clinical practice guidelines and systematic reviews.

## Conclusion

This study provides insight into a primary barrier to EBP among physical therapists – inability to understand and interpret statistics. Clinicians will encounter several hundred statistical terms in the literature. However, just 25 % of the representative terms identified accounted for 90 % of all representative term occurrences. This limited set of representative terms can be used to inform educators designing needed curricula to promote therapists’ skills, competency, and confidence in reading their professional literature. We recommend five strategies for developing such curricula. First, use the common representative terms identified in this study as a foundation for prioritizing what physical therapists need to know. Second, use the categories provided to organize curricula, recognizing that learning will cross categories. Third, use study design frequency and anticipated changes in publication patterns to inform which terms are most important. Fourth, address skills for independent learning of unfamiliar statistical terms. Fifth, develop online references and refresher resources for practicing clinicians. Research is needed to measure the impact of such curricula on physical therapists’ propensity to become evidence based practitioners.

### Ethics approval and consent to participate

Not applicable.

### Consent for publication

Not applicable.

### Availability of data and materials

All data is available through the first author of the manuscript.

## Additional files

Additional file 1: Table with all representative statistical terms and the individual terms that they represent, table with summary findings of terms by study design, and six additional tables showing the most common representative terms for the six most represented study designs. (DOCX 62 kb) Additional file 2: Study design count and frequency for each journal. (DOCX 21 kb)

## Abbreviations

EBP: evidence based practice.
sd: standard deviation
