# Supplementary material for: A bibliometric analysis of statistical terms used in American Physical Therapy Association journals (2011-2012): evidence for educating physical therapists
Source: BMC Med Educ. 2016 Apr 22;16:118. doi: 10.1186/s12909-016-0641-1 (PMC4840969; doi:10.1186/s12909-016-0641-1)
Supplement: Additional file 1: — Table with all representative statistical terms and the individual terms that they represent, table with summary findings of terms by study design, and six additional tables showing the most common representative terms for the six most represented study designs. (DOCX 62 kb) [file 12909_2016_641_MOESM1_ESM.docx]

Additional File 1 – Statistical Terms

[Table 1. All Representative Terms (with terms combined to create them) (n=391 articles) 2](#_Toc438215627)

[Table 2. Summary findings for representative statistical terms by study design 13](#_Toc438215628)

[Table 3. Common Representative Statistical Terms: Cohort Studies (n=127 articles) 14](#_Toc438215629)

[Table 4. Common Representative Statistical Terms: Case Reports (n=66 articles) 17](#_Toc438215630)

[Table 5. Common Representative Statistical Terms: Randomized Controlled Trials (n=31 articles) 18](#_Toc438215631)

[Table 6. Common Representative Statistical Terms: Cross Sectional Studies (n=29 articles) 21](#_Toc438215632)

[Table 7. Common Representative Statistical Terms: Systematic Reviews (n=19 articles) 24](#_Toc438215633)

# Table 1. All Representative Terms (with terms combined to create them) (n=391 articles)

| **All statistical terms (representative term in bold)** | **Number of articles using term** | **Percentage of articles using term** | **Percent of all term occurrences** | **Cumulative Percentage** | **Category** |
| --- | --- | --- | --- | --- | --- |
| **Minimum/Maximum/range** | 327 | 83.6% | 6.4% | 6.4% | Measures of central tendency |
| **Percentage/Proportion**/% | 324 | 82.9% | 6.3% | 12.7% | Measures of central tendency |
| **Mean**/average | 303 | 77.5% | 5.9% | 18.6% | Measures of central tendency |
| **Sample Size** (N) | 263 | 67.3% | 5.1% | 23.7% | Measures of central tendency |
| **Standard Deviation** / SD | 252 | 64.5% | 4.9% | 28.6% | Measures of central tendency |
| **p-value**/p value / p / probability value | 242 | 61.9% | 4.7% | 33.4% | Results terms |
| **Statistical significance**/significant/significance/statistically significant | 194 | 49.6% | 3.8% | 37.1% | Results terms |
| Confidence Intervals (CI) | 136 | 34.8% | 2.7% | 39.8% | Clinically meaningful statistics |
| **Significance level** (α, alpha, corrected alpha value, level of significance) / alpha level / alpha coefficient / Criterion alpha (α) | 125 | 32.0% | 2.4% | 42.2% | Results terms |
| **t-test**: 2-tailed p-values / 2-tailed t test / 2-sided t-test / 2-sided alternative hypothesis / 2-sample t-test /Independent t-test/independent sample t test / independent 1-tailed t-test / independent samples unequal variance t-test / unpaired t-test /Paired t-test/paired sample t-test/repeated groups' paired t test / related samples t-test / 1-sample t-test | 119 | 30.4% | 2.3% | 44.5% | Between Group(s) Comparison |
| **Normal distribution** /normally distributed/ Normalized / Standardized / Normative Data/ Normality | 106 | 27.1% | 2.1% | 46.6% | Measures of central tendency |
| Intraclass Correlation Coefficient ICC | 103 | 26.3% | 2.0% | 48.6% | Measures of association |
| **Descriptive Statistics**/Variables (position and dispersion measurements)/Descriptive Analysis | 100 | 25.6% | 1.9% | 50.6% | Measures of central tendency |
| **Pearson correlation coefficient** / r value/ r-value / Pearson product-moment correlation analysis | 91 | 23.3% | 1.8% | 52.3% | Measures of association |
| **Standard error** / standard error of measure/ standard error of the mean (SEM) / standard error of the estimate | 77 | 19.7% | 1.5% | 53.8% | Measures of central tendency |
| Median | 73 | 18.7% | 1.4% | 55.3% | Measures of central tendency |
| post hoc analysis | 69 | 17.6% | 1.3% | 56.6% | Sundry statistical terms |
| **Between group differences**/between-group/within-group differences/inter group/between-subjects/within-subject | 62 | 15.9% | 1.2% | 57.8% | Between Group(s) Comparison |
| **Chi-Square test** /chi square test / Hosmer-Lemeshow chi square / Pearson Chi-square / χ*2 /* Pearson chi-square test of independence | 61 | 15.6% | 1.2% | 59.0% | Measures of association |
| **F statistic** /F value/F-value / F ratio | 59 | 15.1% | 1.2% | 60.2% | Results terms |
| Independent / Dependent Variable(s) | 58 | 14.8% | 1.1% | 61.3% | Describing variables |
| **Analysis of variance** (**ANOVA)** (basic): 1-Way ANOVA /1-way repeated measures ANOVA / 2 by 2 ANOVA / 2 by 3 ANOVA / 2 by 3 factorial repeated-measure ANOVA /2 way ANOVA / 2 X 2 repeated-measures factorial ANOVA / 2 x 2 x 3 factorial ANOVA / 2 x 2 x6 ANOVA / 2-way multivariate ANOVA / 2-way random effects model ANOVA / 2-way repeated ANOVA / 3-way ANOVA / ANOVA for repeated measures / ANOVA F-type test / linear model for repeated / univariate 1 by 3 ANOVA measures ANOVA / 1-way random effects model / 2-way random effects model / random-effects variable / 2 x 2 x 4 linear mixed model / linear mixed model / 2-way mixed-effects model / 3 factor mixed-model analysis | 56 | 14.3% | 1.1% | 62.4% | Between Group(s) Comparison |
| **Power/Power Analysis** | 55 | 14.1% | 1.1% | 63.5% | Sundry statistical terms |
| Ratio | 51 | 13.0% | 1.0% | 64.4% | Results terms |
| **Sensitivity/Specificity** | 51 | 13.0% | 1.0% | 65.4% | Diagnostic Statistics |
| **parametric / non-parametric**/ nonparametric | 50 | 12.8% | 1.0% | 66.4% | Sundry statistical terms |
| **Mean change scores** / mean change / mean difference | 49 | 12.5% | 1.0% | 67.4% | Measures of central tendency |
| **Minimal Detectable Change** (MDC) / Minimal Detectable Difference (MDD) / Least Detectable Difference (LDD) / Smallest detectable difference (SDDs) / smallest real difference (SRD) / relative value (SRD%) | 49 | 12.5% | 1.0% | 68.3% | Clinically meaningful statistics |
| **Minimal Clinically important Difference** (MCID) / Minimum important difference (MID) / Minimally Important Change (MIC) (MCIC) / Minimal clinically important improvement (MCII) / minimal relevant difference | 48 | 12.3% | 0.9% | 69.3% | Clinically meaningful statistics |
| Effect Size | 45 | 11.5% | 0.9% | 70.1% | Clinically meaningful statistics |
| **Bonferroni Adjustment**/Correction / Bonferroni-Dunn's procedure / bonferroni-holm test / bonferroni holm test | 42 | 10.7% | 0.8% | 71.0% | Between Group(s) Comparison |
| A priori | 41 | 10.5% | 0.8% | 71.8% | Sundry statistical terms |
| **t-score** / t value / t-value / t score /t statistic | 35 | 9.0% | 0.7% | 72.4% | Results terms |
| **ANOVA (repeated measures)**: 2-way repeated ANOVA / 2x2 repeated-measures factorial ANOVA / mixed-effects, repeated measures ANOVA, 2 x 3 factorial repeated-measure ANOVA, lindear model for repeated measures | 34 | 8.7% | 0.7% | 73.1% | Between Group(s) Comparison |
| **Interaction effect** / Interaction / group-time interaction / group by session interaction / group-by-time interaction/time-by-group interaction | 34 | 8.7% | 0.7% | 73.8% | Between Group(s) Comparison |
| Main Effects | 34 | 8.7% | 0.7% | 74.4% | Results terms |
| **Cronbach alpha** / Internal Consistency Coefficient | 32 | 8.2% | 0.6% | 75.0% | Measures of association |
| **Covariate** / covariates / covariation | 32 | 8.2% | 0.6% | 75.7% | Sundry statistical terms |
| **Degrees of freedom** / df | 30 | 7.7% | 0.6% | 76.3% | Sundry statistical terms |
| **Spearman rank correlation** (ρ)/ rho coefficient / Spearman coefficients | 30 | 7.7% | 0.6% | 76.8% | Measures of association |
| Mann-Whitney U test | 29 | 7.4% | 0.6% | 77.4% | Between Group(s) Comparison |
| **r^2^**/R squared/Coefficient of determination | 28 | 7.2% | 0.5% | 78.0% | Results terms |
| Kappa | 28 | 7.2% | 0.5% | 78.5% | Measures of association |
| **Wilcoxon signed rank test** / Wilcoxon T / rank sum test / Wilcoxon matched pairs analysis / Wilcoxon Z | 27 | 6.9% | 0.5% | 79.0% | Between Group(s) Comparison |
| **Interquartile**/Interquartile range (IQR)/quartiles | 27 | 6.9% | 0.5% | 79.6% | Measures of central tendency |
| **Pairwise Comparison** /Pair-wise Correction/ Pairwise error rate (αPC) | 26 | 6.6% | 0.5% | 80.1% | Sundry statistical terms |
| **variance** / variance inflation factor | 26 | 6.6% | 0.5% | 80.6% | Measures of central tendency |
| **z-score** / z score | 26 | 6.6% | 0.5% | 81.1% | Results terms |
| **Regression** / Regression model / Regression Lines / Regressor / Multiple Regression analysis / Multivariable regression / Heirarchical multiple regression / Stepwise multiple regression (forward or backward) | 26 | 6.6% | 0.5% | 81.6% | Measures of association |
| **Continuous** variables / continuous data | 21 | 5.4% | 0.4% | 82.0% | Describing variables |
| **Odds Ratio** / adjusted odds ratio | 21 | 5.4% | 0.4% | 82.4% | Results terms |
| **dichotomous** / dichotomize / dichotomized | 20 | 5.1% | 0.4% | 82.8% | Describing variables |
| **error type (1 or 2)**: Type 1 error/Type 2 error/Type I/Type II | 19 | 4.9% | 0.4% | 83.2% | Sundry statistical terms |
| **Standardized Coeffieicnt (Beta/β)** / Unstandardized Coefficient (Beta/β) | 19 | 4.9% | 0.4% | 83.5% | Measures of association |
| **Analysis of Covariance (ANCOVA)** / 1-way ANCOVA /Univariate ANCOVA / 2-way ANCOVA | 18 | 4.6% | 0.4% | 83.9% | Between Group(s) Comparison |
| Kolmogorov-Smirnov test | 18 | 4.6% | 0.4% | 84.2% | Measures of central tendency |
| **Sample size calculation**/estimate | 18 | 4.6% | 0.4% | 84.6% | Sundry statistical terms |
| Area Under the Curve (AUC) | 17 | 4.3% | 0.3% | 84.9% | Diagnostic Statistics |
| Likelihood ratios | 17 | 4.3% | 0.3% | 85.2% | Clinically meaningful statistics |
| Receiver operator characteristic curve | 17 | 4.3% | 0.3% | 85.6% | Diagnostic Statistics |
| **Logistic regression:** multiple or single-variable logistic regression analysis / multivariate logisitc regression analysis | 17 | 4.3% | 0.3% | 85.9% | Measures of association |
| **Frequency/Contingency Tables** / Frequency Distributions | 15 | 3.8% | 0.3% | 86.2% | Measures of central tendency |
| Multivariate analysis of variance (MANOVA) | 15 | 3.8% | 0.3% | 86.5% | Between Group(s) Comparison |
| **Percent difference** / Percent of change / percent change | 15 | 3.8% | 0.3% | 86.8% | Between Group(s) Comparison |
| Tukey test | 15 | 3.8% | 0.3% | 87.1% | Between Group(s) Comparison |
| Association | 14 | 3.6% | 0.3% | 87.3% | Measures of association |
| **Cohen's d** / d score/ cohen d / d value | 14 | 3.6% | 0.3% | 87.6% | Clinically meaningful statistics |
| Floor/ceiling effect | 14 | 3.6% | 0.3% | 87.9% | Results terms |
| **ordinal** / ordinal variable or data | 13 | 3.3% | 0.3% | 88.1% | Describing variables |
| **Probability** / predictive probability | 13 | 3.3% | 0.3% | 88.4% | Results terms |
| **bivariate analysis** / bivariate / binary variable / bivariate associations / bivariate corrleation / Pearson bivariate analysis | 12 | 3.1% | 0.2% | 88.6% | Measures of association |
| **Categorical** variables | 12 | 3.1% | 0.2% | 88.9% | Describing variables |
| **Linear regression** / multiple linear regression | 12 | 3.1% | 0.2% | 89.1% | Measures of association |
| **Reliability coefficient** / test-retest reliability value | 12 | 3.1% | 0.2% | 89.3% | Results terms |
| **Statistical pooling** / pool / pooling / statistically pooled | 11 | 2.8% | 0.2% | 89.6% | Sundry statistical terms |
| **Coefficient of variation** / Coefficient of variation percentage | 10 | 2.6% | 0.2% | 89.7% | Results terms |
| **Fisher's exact test** / Fisher exact test / Fischer's test | 9 | 2.3% | 0.2% | 89.9% | Measures of association |
| Histogram | 9 | 2.3% | 0.2% | 90.1% | Measures of central tendency |
| **Kruskal-Wallis ANOVA** / Kruskal-Wallis tests | 9 | 2.3% | 0.2% | 90.3% | Between Group(s) Comparison |
| **Median change scores** / Median Difference between groups | 9 | 2.3% | 0.2% | 90.4% | Measures of central tendency |
| Meta-analysis | 9 | 2.3% | 0.2% | 90.6% | Sundry statistical terms |
| multiple comparisons/multiple corrections / multiple t-tests | 8 | 2.0% | 0.2% | 90.8% | N/A here and below |
| Box plot | 7 | 1.8% | 0.1% | 90.9% |  |
| Shapiro-Wilk Test | 7 | 1.8% | 0.1% | 91.1% |  |
| skewed distribution / not normally distributed / skewness | 7 | 1.8% | 0.1% | 91.2% |  |
| Standardized Response Mean SRM | 7 | 1.8% | 0.1% | 91.3% |  |
| Themes | 7 | 1.8% | 0.1% | 91.5% |  |
| Wilk's lambda / wilks | 7 | 1.8% | 0.1% | 91.6% |  |
| ANOVA (Mixed deisgns): Mixed Effects (second-level, first-level) / mixed effects model / Mixed Effects ANOVA / mixed-effects, repeated-measures ANOVA | 6 | 1.5% | 0.1% | 91.7% |  |
| Confounding variable / confounders | 6 | 1.5% | 0.1% | 91.8% |  |
| homoscedasticity / homogeneity of variance / homogeneity | 6 | 1.5% | 0.1% | 91.9% |  |
| Percentage Agreement | 6 | 1.5% | 0.1% | 92.1% |  |
| Root mean square (RMS) error | 6 | 1.5% | 0.1% | 92.2% |  |
| ANOVA-by-Rank Test/Friedman's ANOVA | 5 | 1.3% | 0.1% | 92.3% |  |
| Bland-Altman plot | 5 | 1.3% | 0.1% | 92.4% |  |
| Clusters / cluster option | 5 | 1.3% | 0.1% | 92.5% |  |
| dispersion | 5 | 1.3% | 0.1% | 92.6% |  |
| factor analysis / exploratory factor analysis / confirmatory factor analysis / factor analysis | 5 | 1.3% | 0.1% | 92.7% |  |
| Friedman rank test | 5 | 1.3% | 0.1% | 92.8% |  |
| Greenhouse-Geisser Correction | 5 | 1.3% | 0.1% | 92.9% |  |
| Multivariate analysis of covariance (MANCOVA) | 5 | 1.3% | 0.1% | 93.0% |  |
| regression coefficient / standardized regression coefficient | 5 | 1.3% | 0.1% | 93.1% |  |
| Relative Risk / risk ratio / adjusted risk ratio | 5 | 1.3% | 0.1% | 93.2% |  |
| univariate relationship / univariate tests | 5 | 1.3% | 0.1% | 93.3% |  |
| Bootstrap method / boot-strapping | 4 | 1.0% | 0.1% | 93.3% |  |
| Cohen interrater kappa value | 4 | 1.0% | 0.1% | 93.4% |  |
| Collinearity / collinear / colinearity | 4 | 1.0% | 0.1% | 93.5% |  |
| Forest plot | 4 | 1.0% | 0.1% | 93.6% |  |
| Free Marginal Kappa Coefficient | 4 | 1.0% | 0.1% | 93.6% |  |
| homogenous / heterogenous / heterogeneity | 4 | 1.0% | 0.1% | 93.7% |  |
| Inferential Analysis / inferential statistics | 4 | 1.0% | 0.1% | 93.8% |  |
| intention to treat/ intent to treat | 4 | 1.0% | 0.1% | 93.9% |  |
| least square error / least squares mean | 4 | 1.0% | 0.1% | 94.0% |  |
| Limits of Agreement (LOA) / LA | 4 | 1.0% | 0.1% | 94.0% |  |
| logit / logit value | 4 | 1.0% | 0.1% | 94.1% |  |
| McNemar Test/ McNemar-Bowker test / McNemar pair analysis | 4 | 1.0% | 0.1% | 94.2% |  |
| Mean squared error | 4 | 1.0% | 0.1% | 94.3% |  |
| Nagelkerke r^2^ | 4 | 1.0% | 0.1% | 94.3% |  |
| Natural log transformations | 4 | 1.0% | 0.1% | 94.4% |  |
| nominal variable(s) | 4 | 1.0% | 0.1% | 94.5% |  |
| Number needed to treat (NNT/NND) | 4 | 1.0% | 0.1% | 94.6% |  |
| Scatter Plot / Scatter Gram | 4 | 1.0% | 0.1% | 94.7% |  |
| triangulation | 4 | 1.0% | 0.1% | 94.7% |  |
| Absolute Error (AE) | 3 | 0.8% | 0.1% | 94.8% |  |
| Absolute risk reduction (ARR) | 3 | 0.8% | 0.1% | 94.9% |  |
| Comparative fit index (CFI) | 3 | 0.8% | 0.1% | 94.9% |  |
| eta squared / partial eta squared | 3 | 0.8% | 0.1% | 95.0% |  |
| Kaplan-Meier estimator / Kaplain-Meier survival analysis | 3 | 0.8% | 0.1% | 95.0% |  |
| Levene's test for equality of variances / Levene's test for homogeneity | 3 | 0.8% | 0.1% | 95.1% |  |
| Mauchly test / assumption of sphericity / Mauchly's sphericity test | 3 | 0.8% | 0.1% | 95.1% |  |
| Member Checking | 3 | 0.8% | 0.1% | 95.2% |  |
| Mode | 3 | 0.8% | 0.1% | 95.3% |  |
| Multicollinearity | 3 | 0.8% | 0.1% | 95.3% |  |
| negative predictive value / positive predictive value | 3 | 0.8% | 0.1% | 95.4% |  |
| point biserial correlation/biserial correlation method | 3 | 0.8% | 0.1% | 95.4% |  |
| Quadratic-weighted kappa coefficients/weighted kappa | 3 | 0.8% | 0.1% | 95.5% |  |
| responsiveness | 3 | 0.8% | 0.1% | 95.6% |  |
| Stepwise backward regression model / stepwise forward regression model | 3 | 0.8% | 0.1% | 95.6% |  |
| Type 3 sum of squares/ sum of squares / type 1 sum of squares model | 3 | 0.8% | 0.1% | 95.7% |  |
| U value / U score | 3 | 0.8% | 0.1% | 95.7% |  |
| varimax rotation | 3 | 0.8% | 0.1% | 95.8% |  |
| weighted mean difference (WMD) / weighted standardized mean difference (WSMD) | 3 | 0.8% | 0.1% | 95.8% |  |
| binary logical regression analysis | 2 | 0.5% | 0.0% | 95.9% |  |
| Cell means contrasts / contrast comparison | 2 | 0.5% | 0.0% | 95.9% |  |
| clinically meaningful difference / clinically meaningful change | 2 | 0.5% | 0.0% | 96.0% |  |
| delphi technique | 2 | 0.5% | 0.0% | 96.0% |  |
| differential item functioning | 2 | 0.5% | 0.0% | 96.0% |  |
| Eigenvalue | 2 | 0.5% | 0.0% | 96.1% |  |
| family-wise alpha / family wise alpha / family wise alpha adjustment | 2 | 0.5% | 0.0% | 96.1% |  |
| Group Effects | 2 | 0.5% | 0.0% | 96.2% |  |
| Guyatt responsiveness ratio (GRR) / Guyatt responsiveness index (GRI) | 2 | 0.5% | 0.0% | 96.2% |  |
| Hotelling T2 test / Hotelling T squared test / Test information function | 2 | 0.5% | 0.0% | 96.2% |  |
| I squared statistic / I squared | 2 | 0.5% | 0.0% | 96.3% |  |
| imputation technique | 2 | 0.5% | 0.0% | 96.3% |  |
| Item response theory method | 2 | 0.5% | 0.0% | 96.4% |  |
| Kurtosis | 2 | 0.5% | 0.0% | 96.4% |  |
| log likelihood | 2 | 0.5% | 0.0% | 96.4% |  |
| log-transformed/log transformed | 2 | 0.5% | 0.0% | 96.5% |  |
| maximum-likelihood method / maximum likelihood extraction/estimate (MLE) | 2 | 0.5% | 0.0% | 96.5% |  |
| Mean Effect size (g value)/g value / Mean Effect | 2 | 0.5% | 0.0% | 96.5% |  |
| Monte Carlo Test | 2 | 0.5% | 0.0% | 96.6% |  |
| ordinary least squares regression | 2 | 0.5% | 0.0% | 96.6% |  |
| parsimonious isometric multivariate model / parsimonious model | 2 | 0.5% | 0.0% | 96.7% |  |
| Partial Correlation | 2 | 0.5% | 0.0% | 96.7% |  |
| predictor-variable | 2 | 0.5% | 0.0% | 96.7% |  |
| Rasch analysis | 2 | 0.5% | 0.0% | 96.8% |  |
| Repeated Measures Effect | 2 | 0.5% | 0.0% | 96.8% |  |
| Risk-adjusted prediction models / risk adjusted multivariable predictive model | 2 | 0.5% | 0.0% | 96.9% |  |
| Saturation | 2 | 0.5% | 0.0% | 96.9% |  |
| Scatter plot (Blant-Altman plot) | 2 | 0.5% | 0.0% | 96.9% |  |
| Scheffe test / scheffe post hoc test | 2 | 0.5% | 0.0% | 97.0% |  |
| standardized coefficients/ t ratio | 2 | 0.5% | 0.0% | 97.0% |  |
| standardized mean difference (SMD) | 2 | 0.5% | 0.0% | 97.1% |  |
| Step-wise logistic regression | 2 | 0.5% | 0.0% | 97.1% |  |
| Wald Test | 2 | 0.5% | 0.0% | 97.1% |  |
| wilson score | 2 | 0.5% | 0.0% | 97.2% |  |
| Two standard deviation band method | 1 | 0.3% | 0.0% | 97.2% |  |
| absolute value suppression | 1 | 0.3% | 0.0% | 97.2% |  |
| adjusted mean difference(s) | 1 | 0.3% | 0.0% | 97.2% |  |
| Alpha Inflation | 1 | 0.3% | 0.0% | 97.3% |  |
| Analysis of the fit | 1 | 0.3% | 0.0% | 97.3% |  |
| anchor based method | 1 | 0.3% | 0.0% | 97.3% |  |
| Asymptotic power | 1 | 0.3% | 0.0% | 97.3% |  |
| Bartlett Test of Sphericity | 1 | 0.3% | 0.0% | 97.3% |  |
| Bayesian probability | 1 | 0.3% | 0.0% | 97.3% |  |
| best-fit line | 1 | 0.3% | 0.0% | 97.4% |  |
| bivariate scatter plots | 1 | 0.3% | 0.0% | 97.4% |  |
| Central Tendency | 1 | 0.3% | 0.0% | 97.4% |  |
| change coefficient | 1 | 0.3% | 0.0% | 97.4% |  |
| Clopper-Pearson method | 1 | 0.3% | 0.0% | 97.4% |  |
| Cochran Q test of heterogeneity | 1 | 0.3% | 0.0% | 97.5% |  |
| coefficient of agreement | 1 | 0.3% | 0.0% | 97.5% |  |
| coefficient of multiple correlations (CMC) | 1 | 0.3% | 0.0% | 97.5% |  |
| Cohen's power analytic approach | 1 | 0.3% | 0.0% | 97.5% |  |
| compound symmetry covariance structure | 1 | 0.3% | 0.0% | 97.5% |  |
| concordance correlation coefficient | 1 | 0.3% | 0.0% | 97.6% |  |
| constant comparative analysis | 1 | 0.3% | 0.0% | 97.6% |  |
| Content Analysis Method | 1 | 0.3% | 0.0% | 97.6% |  |
| Contrast profile test | 1 | 0.3% | 0.0% | 97.6% |  |
| Control event rate (CER) | 1 | 0.3% | 0.0% | 97.6% |  |
| correlation matrix | 1 | 0.3% | 0.0% | 97.7% |  |
| correlation matrix Kaiser-Meyer-Olkin values | 1 | 0.3% | 0.0% | 97.7% |  |
| covariate-adjusted effect | 1 | 0.3% | 0.0% | 97.7% |  |
| Cox proportional hazard regression | 1 | 0.3% | 0.0% | 97.7% |  |
| Cross Correlation Coefficients | 1 | 0.3% | 0.0% | 97.7% |  |
| Cross product | 1 | 0.3% | 0.0% | 97.8% |  |
| cross-loading | 1 | 0.3% | 0.0% | 97.8% |  |
| cross-tabulation/crosstabs | 1 | 0.3% | 0.0% | 97.8% |  |
| DeLong's test | 1 | 0.3% | 0.0% | 97.8% |  |
| Derivitave | 1 | 0.3% | 0.0% | 97.8% |  |
| DIF contrast | 1 | 0.3% | 0.0% | 97.9% |  |
| Domain | 1 | 0.3% | 0.0% | 97.9% |  |
| Dot Graph | 1 | 0.3% | 0.0% | 97.9% |  |
| Duncan multiple range test | 1 | 0.3% | 0.0% | 97.9% |  |
| Dunn's pair-wise comparisons | 1 | 0.3% | 0.0% | 97.9% |  |
| error mean square | 1 | 0.3% | 0.0% | 98.0% |  |
| Error variance | 1 | 0.3% | 0.0% | 98.0% |  |
| Experimental event rate (EER) | 1 | 0.3% | 0.0% | 98.0% |  |
| factor extraction | 1 | 0.3% | 0.0% | 98.0% |  |
| false discovery rate algorithm | 1 | 0.3% | 0.0% | 98.0% |  |
| Fisher least significant difference test | 1 | 0.3% | 0.0% | 98.1% |  |
| Fisher r-to-z transformation / Fisher transformation | 1 | 0.3% | 0.0% | 98.1% |  |
| fixed effects | 1 | 0.3% | 0.0% | 98.1% |  |
| fixed marginal kappa | 1 | 0.3% | 0.0% | 98.1% |  |
| forced one-factor solution | 1 | 0.3% | 0.0% | 98.1% |  |
| Freedman method | 1 | 0.3% | 0.0% | 98.1% |  |
| Full-factorial model | 1 | 0.3% | 0.0% | 98.2% |  |
| G statistic/ G-test / log-likelihood chi-square statistic | 1 | 0.3% | 0.0% | 98.2% |  |
| Games-Howell post hoc test | 1 | 0.3% | 0.0% | 98.2% |  |
| general linear regression model (GLM) | 1 | 0.3% | 0.0% | 98.2% |  |
| Generalized estimating equations GEE | 1 | 0.3% | 0.0% | 98.2% |  |
| Guttman split-half coefficient | 1 | 0.3% | 0.0% | 98.3% |  |
| Hazard Ratio | 1 | 0.3% | 0.0% | 98.3% |  |
| Hodges-Lehman estimates | 1 | 0.3% | 0.0% | 98.3% |  |
| Huber-White sandwich estimator of variance | 1 | 0.3% | 0.0% | 98.3% |  |
| Independent t-contrasts | 1 | 0.3% | 0.0% | 98.3% |  |
| Interobserver agreement | 1 | 0.3% | 0.0% | 98.4% |  |
| Interobserver Percent Agreement | 1 | 0.3% | 0.0% | 98.4% |  |
| interpolating / interpolate | 1 | 0.3% | 0.0% | 98.4% |  |
| intersubject variability | 1 | 0.3% | 0.0% | 98.4% |  |
| inverse probability weights | 1 | 0.3% | 0.0% | 98.4% |  |
| inverse transformation | 1 | 0.3% | 0.0% | 98.5% |  |
| Inverted U-Shaped Curve Theory | 1 | 0.3% | 0.0% | 98.5% |  |
| jackknife residual/jackknife leverage | 1 | 0.3% | 0.0% | 98.5% |  |
| Kaiser-Guttmann rule | 1 | 0.3% | 0.0% | 98.5% |  |
| Kendall W | 1 | 0.3% | 0.0% | 98.5% |  |
| last-observation-carried-forward (LOCF) | 1 | 0.3% | 0.0% | 98.6% |  |
| latent variable | 1 | 0.3% | 0.0% | 98.6% |  |
| Lilliefors Significance Correction | 1 | 0.3% | 0.0% | 98.6% |  |
| linear contrast | 1 | 0.3% | 0.0% | 98.6% |  |
| linear discriminant analysis (LDA) | 1 | 0.3% | 0.0% | 98.6% |  |
| Linear transformation/ nonlinear Transformation | 1 | 0.3% | 0.0% | 98.7% |  |
| Linearity | 1 | 0.3% | 0.0% | 98.7% |  |
| Log rank test | 1 | 0.3% | 0.0% | 98.7% |  |
| Logistic Coefficient *b* | 1 | 0.3% | 0.0% | 98.7% |  |
| Lyapunov Exponent calculation (LyE) | 1 | 0.3% | 0.0% | 98.7% |  |
| McFadden rho-squared value | 1 | 0.3% | 0.0% | 98.8% |  |
| mean comparisons | 1 | 0.3% | 0.0% | 98.8% |  |
| mean slope of change | 1 | 0.3% | 0.0% | 98.8% |  |
| mean square contingency coefficient / phi coefficient | 1 | 0.3% | 0.0% | 98.8% |  |
| mediation model | 1 | 0.3% | 0.0% | 98.8% |  |
| Minimum voxel extent (k) / Voxel | 1 | 0.3% | 0.0% | 98.8% |  |
| Mixed-factor ANOVA | 1 | 0.3% | 0.0% | 98.9% |  |
| Multivariate Assumptions | 1 | 0.3% | 0.0% | 98.9% |  |
| multivariate effects | 1 | 0.3% | 0.0% | 98.9% |  |
| multivariate model | 1 | 0.3% | 0.0% | 98.9% |  |
| multivariate repeated-measures mixed-effects model | 1 | 0.3% | 0.0% | 98.9% |  |
| Nagelkerke r^2 | 1 | 0.3% | 0.0% | 99.0% |  |
| Natural log of the odds | 1 | 0.3% | 0.0% | 99.0% |  |
| Newman-Keuls post hoc comparison | 1 | 0.3% | 0.0% | 99.0% |  |
| normal fit index | 1 | 0.3% | 0.0% | 99.0% |  |
| normality plots | 1 | 0.3% | 0.0% | 99.0% |  |
| Omnibus Test / Omnibus ANOVA | 1 | 0.3% | 0.0% | 99.1% |  |
| path analysis | 1 | 0.3% | 0.0% | 99.1% |  |
| Pitman test | 1 | 0.3% | 0.0% | 99.1% |  |
| Poisson distribution model | 1 | 0.3% | 0.0% | 99.1% |  |
| polynomial regression line | 1 | 0.3% | 0.0% | 99.1% |  |
| prediction model | 1 | 0.3% | 0.0% | 99.2% |  |
| Pre-planned/planned contrasts | 1 | 0.3% | 0.0% | 99.2% |  |
| prevalence estimate (p) | 1 | 0.3% | 0.0% | 99.2% |  |
| prevalence-adjusted and bias-adjusted kappa (PABAK) coefficient | 1 | 0.3% | 0.0% | 99.2% |  |
| Principal components analysis | 1 | 0.3% | 0.0% | 99.2% |  |
| probability plot | 1 | 0.3% | 0.0% | 99.3% |  |
| qualitative analysis | 1 | 0.3% | 0.0% | 99.3% |  |
| radar graph | 1 | 0.3% | 0.0% | 99.3% |  |
| random coefficient model | 1 | 0.3% | 0.0% | 99.3% |  |
| Random Effect | 1 | 0.3% | 0.0% | 99.3% |  |
| Random Pair Effect | 1 | 0.3% | 0.0% | 99.4% |  |
| random-intercept model / multilevel modeling | 1 | 0.3% | 0.0% | 99.4% |  |
| relative postion measure | 1 | 0.3% | 0.0% | 99.4% |  |
| residual correlation | 1 | 0.3% | 0.0% | 99.4% |  |
| residual variance | 1 | 0.3% | 0.0% | 99.4% |  |
| scree plot | 1 | 0.3% | 0.0% | 99.5% |  |
| semiquartile | 1 | 0.3% | 0.0% | 99.5% |  |
| sidak correction | 1 | 0.3% | 0.0% | 99.5% |  |
| significant correlation (2-tailed) | 1 | 0.3% | 0.0% | 99.5% |  |
| simple effects | 1 | 0.3% | 0.0% | 99.5% |  |
| single-subject contrast map | 1 | 0.3% | 0.0% | 99.6% |  |
| smallest real difference (SRD) / relative value (SRD%) | 1 | 0.3% | 0.0% | 99.6% |  |
| sobel test | 1 | 0.3% | 0.0% | 99.6% |  |
| spearman-brown techniques | 1 | 0.3% | 0.0% | 99.6% |  |
| sphericity | 1 | 0.3% | 0.0% | 99.6% |  |
| squared type II semi-partial r^2^ coefficients | 1 | 0.3% | 0.0% | 99.6% |  |
| square-root transformation/ square root transformation | 1 | 0.3% | 0.0% | 99.7% |  |
| Standardized root mean square residual (SRMR) | 1 | 0.3% | 0.0% | 99.7% |  |
| stochastic independence | 1 | 0.3% | 0.0% | 99.7% |  |
| structural equation modeling | 1 | 0.3% | 0.0% | 99.7% |  |
| structural variability | 1 | 0.3% | 0.0% | 99.7% |  |
| surrogation analysis | 1 | 0.3% | 0.0% | 99.8% |  |
| T2 / t square value | 1 | 0.3% | 0.0% | 99.8% |  |
| Test information function | 1 | 0.3% | 0.0% | 99.8% |  |
| Theta (θ) Scores | 1 | 0.3% | 0.0% | 99.8% |  |
| time-dependent effects | 1 | 0.3% | 0.0% | 99.8% |  |
| Transformed Ranks/ransformed into ranks | 1 | 0.3% | 0.0% | 99.9% |  |
| Trucker-Lewis Index (TLI) | 1 | 0.3% | 0.0% | 99.9% |  |
| Variable Error (VE) | 1 | 0.3% | 0.0% | 99.9% |  |
| Woolf-Haldane correction | 1 | 0.3% | 0.0% | 99.9% |  |
| Yates correction | 1 | 0.3% | 0.0% | 99.9% |  |
| youden index | 1 | 0.3% | 0.0% | 100.0% |  |
| zero-order analysis | 1 | 0.3% | 0.0% | 100.0% |  |
| zero-order mean comparisons | 1 | 0.3% | 0.0% | 100.0% |  |
|  | 5130 |  | 100.00% |  |  |

# Table 2. Summary findings for representative statistical terms by study design

| **Study Design** | **Number of Articles** | **Terms/article (SD)** | **Total number of terms** | **Number of terms representing 90% of all occurrences** |
| --- | --- | --- | --- | --- |
| Prospective cohort | 127 | 15.9(6.1) | 212 | 74 |
| Case report | 66 | 3.6(2.9) | 47 | 24 |
| Randomized controlled trial | 31 | 19.3(6.2) | 120 | 64 |
| Cross sectional | 29 | 19.6(5.7) | 119 | 68 |
| Systematic review | 19 | 12.2(6.7) | 74 | 52 |
| **All designs (n=21)** | **391** | **13.1(8.0)** | **321** | **81** |

* Only the 5 most common study designs are listed.

# Table 3. Common Representative Statistical Terms: Cohort Studies (n=127 articles)

|  | **Number of articles using term** | **Percentage of articles using term** | **Percent of all term occurrences** | **Cumulative Percentage** |
| --- | --- | --- | --- | --- |
| Mean/average | 119 | 93.7% | 5.9% | 5.9% |
| Minimum/maximum/range | 112 | 88.2% | 5.6% | 11.5% |
| Standard Deviation / SD | 110 | 86.6% | 5.5% | 16.9% |
| P-Value/p value / p / probability value | 108 | 85.0% | 5.4% | 22.3% |
| Sample Size (N) | 106 | 83.5% | 5.3% | 27.6% |
| Percentage/Proportion/% | 104 | 81.9% | 5.2% | 32.7% |
| Statistical significance/difference/significant/significance/statistically significant | 84 | 66.1% | 4.2% | 36.9% |
| T-tests: 2-tailed p-values / 2-tailed t test / 2-sided t-test / 2-sided alternative hypothesis / 2-sample t-test /Independent t-test/independent sample t test / independent 1-tailed t-test / independent samples unequal variance t-test / unpaired t-test /Paired t-test/paired sample t-test/repeated groups' paired t test / related samples t-test / 1-sample t-test / student t-test / t statistic | 56 | 44.1% | 2.8% | 39.7% |
| Confidence Intervals (CI) | 53 | 41.7% | 2.6% | 42.3% |
| Significance level (α, alpha, corrected alpha value, level of significance) / alpha level / alpha coefficient / Criterion alpha (α) | 52 | 40.9% | 2.6% | 44.9% |
| Normally distributed/normal distribution / Normalized / Standardized / Normative Data/ Normality | 42 | 33.1% | 2.1% | 47.0% |
| Descriptive Statistics/Variables (position and dispersion measurements)/Descriptive Analysis | 39 | 30.7% | 1.9% | 48.9% |
| Intraclass Correlation Coefficient ICC | 38 | 29.9% | 1.9% | 50.8% |
| Pearson correlation coefficient r value/ r-value / Pearson product-moment correlation analysis | 37 | 29.1% | 1.8% | 52.7% |
| post hoc analysis | 36 | 28.3% | 1.8% | 54.4% |
| Standard Error / Standard Error of Measure/ Standard Error of the Mean (SEM) / Standard Error of the Estimate | 32 | 25.2% | 1.6% | 56.0% |
| Median | 29 | 22.8% | 1.4% | 57.5% |
| F value/F-value / f statistic / F ratio | 25 | 19.7% | 1.2% | 58.7% |
| ANOVA (basic): 1-Way ANOVA /1-way repeated measures ANOVA / 2 by 2 ANOVA / 2 by 3 ANOVA / 2 by 3 factorial repeated-measure ANOVA /2 way ANOVA / 2 X 2 repeated-measures factorial ANOVA / 2 x 2 x 3 factorial ANOVA / 2 x 2 x6 ANOVA / 2-way multivariate ANOVA / 2-way random effects model ANOVA / 2-way repeated ANOVA / 3-way ANOVA / ANOVA for repeated measures / ANOVA F-type test / linear model for repeated / univariate 1 by 3 ANOVA measures ANOVA / 1-way random effects model / 2-way random effects model / random-effects variable / 2 x 2 x 4 linear mixed model / linear mixed model / 2-way mixed-effects model / 3 factor mixed-model analysis | 25 | 19.7% | 1.2% | 60.0% |
| non-parametric/ nonparametric / parametric | 25 | 19.7% | 1.2% | 61.2% |
| Between group differences/between-group/within-group differences/inter group/between-subjects/within-subject | 24 | 18.9% | 1.2% | 62.4% |
| Chi-Square test/chi square test / Hosmer-Lemeshow chi square / Pearson Chi-square / *X(2) / Pearson chi-square test of independence* | 22 | 17.3% | 1.1% | 63.5% |
| A priori | 21 | 16.5% | 1.0% | 64.5% |
| Power/Power Analysis | 21 | 16.5% | 1.0% | 65.6% |
| Mean change scores / mean change / mean difference | 19 | 15.0% | 0.9% | 66.5% |
| Independent / Dependent Variable(s) | 18 | 14.2% | 0.9% | 67.4% |
| Wilcoxon signed rank test / Wilcoxon T / rank sum test / Wilcoxon matched pairs analysis / Wilcoxon Z | 18 | 14.2% | 0.9% | 68.3% |
| t value/t-value/t-score/t score/t statistic | 17 | 13.4% | 0.8% | 69.2% |
| Bonferroni Adjustment/Correction / Bonferroni-Dunn's procedure / bonferroni-holm test / bonferroni holm test | 16 | 12.6% | 0.8% | 69.9% |
| ANOVA (repeated measures): 2-way repeated ANOVA / 2x2 repeated-measures factorial ANOVA / mixed-effects, repeated measures ANOVA, 2 x 3 factorial repeated-measure ANOVA, lindear model for repeated measures | 16 | 12.6% | 0.8% | 70.7% |
| Main Effects | 16 | 12.6% | 0.8% | 71.5% |
| Other Ratio | 15 | 11.8% | 0.7% | 72.3% |
| Effect Size | 15 | 11.8% | 0.7% | 73.0% |
| Spearman Coefficients/spearman rank correlation (ρ)/ rho coefficient | 15 | 11.8% | 0.7% | 73.8% |
| Pairwise Comparison /Pair-wise Correction/ Pairwise error rate (αPC) | 15 | 11.8% | 0.7% | 74.5% |
| Sensitivity/Specificity | 14 | 11.0% | 0.7% | 75.2% |
| Interaction / Interaction effect / group-time interaction / group by session interaction / group-by-time interaction/time-by-group interaction | 14 | 11.0% | 0.7% | 75.9% |
| Interquartile/Interquartile range (IQR)/quartiles | 14 | 11.0% | 0.7% | 76.6% |
| Minimal Detectable Change (MDC) / Minimal Detectable Difference (MDD) / Least Detectable Difference (LDD) / Smallest detectable difference (SDDs) / smallest real difference (SRD) / relative value (SRD%) | 13 | 10.2% | 0.6% | 77.2% |
| Covariate / covariates / covariation | 13 | 10.2% | 0.6% | 77.9% |
| Regression analysis / Regression model / Regression Lines / Regressor / Multiple Regression / Multivariable regression / Heirarchical multiple regression / Stepwise multiple regression (forward or backward) | 13 | 10.2% | 0.6% | 78.5% |
| z-score/z score | 13 | 10.2% | 0.6% | 79.2% |
| Mann-Whitney U test | 12 | 9.4% | 0.6% | 79.8% |
| Minimal Clinically important Difference (MCID) / Minimum important difference (MID) / Minimally Important Change (MIC) (MCIC) / Minimal clinically important improvement (MCII) / minimal relevant difference | 11 | 8.7% | 0.5% | 80.3% |
| Degree / Degrees of freedom / df | 12 | 9.4% | 0.6% | 80.9% |
| Variance Inflation Factor / variance | 11 | 8.7% | 0.5% | 81.5% |
| Chronbach alpha / Cronbach alpha / Internal Consistency Coefficient | 10 | 7.9% | 0.5% | 82.0% |
| r^2^/r squared/Coefficient of determination | 10 | 7.9% | 0.5% | 82.5% |
| Type 1 error/Type 2 error/Type I/Type II | 10 | 7.9% | 0.5% | 83.0% |
| Analysis of Covariance (ANCOVA) / 1-way ANCOVA /Univariate ANCOVA / 2-way ANCOVA | 9 | 7.1% | 0.4% | 83.4% |
| Kolmogorov-Smirnov test | 9 | 7.1% | 0.4% | 83.9% |
| Tukey | 9 | 7.1% | 0.4% | 84.3% |
| Kappa | 8 | 6.3% | 0.4% | 84.7% |
| Odds Ratio / adjusted odds ratio | 7 | 5.5% | 0.3% | 85.0% |
| Sample size calculation/estimate | 7 | 5.5% | 0.3% | 85.4% |
| Multivariate analysis of variance (ANOVA) / Multiple analyses of variance (MANOVA) | 7 | 5.5% | 0.3% | 85.7% |
| Percent of change / percent change / percent difference | 7 | 5.5% | 0.3% | 86.1% |
| Continuous variables / continuous data | 6 | 4.7% | 0.3% | 86.4% |
| dichotomize / dichotomous / dichotomized | 6 | 4.7% | 0.3% | 86.7% |
| Unstandardized Coefficient (B/b) / Standardized Coefficient (Beta/β) | 6 | 4.7% | 0.3% | 87.0% |
| ROC curve | 6 | 4.7% | 0.3% | 87.3% |
| Coefficient of variation %/ CV | 6 | 4.7% | 0.3% | 87.6% |
| Median Change Scores / Median Difference between groups | 6 | 4.7% | 0.3% | 87.9% |
| Area Under the Curve (AUC) | 5 | 3.9% | 0.2% | 88.1% |
| Likelihood ratios | 5 | 3.9% | 0.2% | 88.4% |
| multiple or single-variable logistic regression analysis / multivariate logisitc regression analysis | 5 | 3.9% | 0.2% | 88.6% |
| Association | 5 | 3.9% | 0.2% | 88.9% |
| Cohen's d/d score/ cohen d/ d value | 5 | 3.9% | 0.2% | 89.1% |
| Shapiro-Wilk Test | 5 | 3.9% | 0.2% | 89.4% |

Another 143 terms were used 4 or fewer times.

# Table 4. Common Representative Statistical Terms: Case Reports (n=66 articles)

|  | **Number of articles using term** | **Percentage of articles using term** | **Percent of all term occurrences** | **Cumulative Percentage** |
| --- | --- | --- | --- | --- |
| Percentage/Proportion/% | 54 | 81.8% | 22.6% | 22.6% |
| Minimum/Maximum/range | 45 | 68.2% | 18.8% | 41.4% |
| Mean/average | 26 | 39.4% | 10.9% | 52.3% |
| Other Ratio | 11 | 16.7% | 4.6% | 56.9% |
| Sensitivity/Specificity | 9 | 13.6% | 3.8% | 60.7% |
| Intraclass Correlation Coefficient ICC | 8 | 12.1% | 3.3% | 64.0% |
| Minimal Detectable Change (MDC) / Minimal Detectable Difference (MDD) / Least Detectable Difference (LDD) / Smallest detectable difference (SDDs) / smallest real difference (SRD) / relative value (SRD%) | 8 | 12.1% | 3.3% | 67.4% |
| Minimal Clinically important Difference (MCID) / Minimum important difference (MID) / Minimally Important Change (MIC) (MCIC) / Minimal clinically important improvement (MCII) / minimal relevant difference | 8 | 12.1% | 3.3% | 70.7% |
| Standard Deviation / SD | 7 | 10.6% | 2.9% | 73.6% |
| Statistical significance/difference/significant/significance/statistically significant | 6 | 9.1% | 2.5% | 76.2% |
| Normally distributed/normal distribution / Normalized / Standardized / Normative Data/ Normality | 5 | 7.6% | 2.1% | 78.2% |
| Sample Size (N) | 4 | 6.1% | 1.7% | 79.9% |
| Pearson correlation coefficient r value/ r-value / Pearson product-moment correlation analysis | 4 | 6.1% | 1.7% | 81.6% |
| Reliability coefficient / test-retest reliability value | 3 | 4.5% | 1.3% | 82.8% |
| Significance level (α, alpha, corrected alpha value, level of significance) / alpha level / alpha coefficient / Criterion alpha (α) | 2 | 3.0% | 0.8% | 83.7% |
| Descriptive Statistics/Variables (position and dispersion measurements)/Descriptive Analysis | 2 | 3.0% | 0.8% | 84.5% |
| Median | 2 | 3.0% | 0.8% | 85.4% |
| Chronbach alpha / Cronbach alpha / Internal Consistency Coefficient | 2 | 3.0% | 0.8% | 86.2% |
| Kappa | 2 | 3.0% | 0.8% | 87.0% |
| z-score/z score | 2 | 3.0% | 0.8% | 87.9% |
| Percent of change / percent change / percent difference | 2 | 3.0% | 0.8% | 88.7% |
| negative predictive value / positive predictive value | 2 | 3.0% | 0.8% | 89.5% |

Another 25 terms were used just once.

# Table 5. Common Representative Statistical Terms: Randomized Controlled Trials (n=31 articles)

|  | **Number of articles using term** | **Percentage of articles using term** | **Percent of all term occurrences** | **Cumulative Percentage** |
| --- | --- | --- | --- | --- |
| Mean/average | 30 | 96.8% | 4.9% | 4.9% |
| Sample Size (N) | 29 | 93.5% | 4.8% | 9.7% |
| Standard Deviation / SD | 28 | 90.3% | 4.6% | 14.3% |
| P-Value/p value / p / probability value | 28 | 90.3% | 4.6% | 18.9% |
| Minimum/Maximum/range | 28 | 90.3% | 4.6% | 23.5% |
| Percentage/Proportion/% | 27 | 87.1% | 4.4% | 28.0% |
| Statistical significance/difference/significant/significance/statistically significant | 21 | 67.7% | 3.5% | 31.4% |
| T-tests: 2-tailed p-values / 2-tailed t test / 2-sided t-test / 2-sided alternative hypothesis / 2-sample t-test /Independent t-test/independent sample t test / independent 1-tailed t-test / independent samples unequal variance t-test / unpaired t-test /Paired t-test/paired sample t-test/repeated groups' paired t test / related samples t-test / 1-sample t-test / student t-test / t statistic | 19 | 61.3% | 3.1% | 34.5% |
| Confidence Intervals (CI) | 18 | 58.1% | 3.0% | 37.5% |
| Significance level (α, alpha, corrected alpha value, level of significance) / alpha level / alpha coefficient / Criterion alpha (α) | 18 | 58.1% | 3.0% | 40.5% |
| Power/Power Analysis | 16 | 51.6% | 2.6% | 43.1% |
| Normally distributed/normal distribution / Normalized / Standardized / Normative Data/ Normality | 12 | 38.7% | 2.0% | 45.1% |
| Effect Size | 11 | 35.5% | 1.8% | 46.9% |
| Bonferroni Adjustment/Correction / Bonferroni-Dunn's procedure / bonferroni-holm test / bonferroni holm test | 11 | 35.5% | 1.8% | 48.7% |
| Between group differences/between-group/within-group differences/inter group/between-subjects/within-subject | 11 | 35.5% | 1.8% | 50.5% |
| F value/F-value / f statistic / F ratio | 11 | 35.5% | 1.8% | 52.3% |
| post hoc analysis | 10 | 32.3% | 1.6% | 53.9% |
| Minimal Clinically important Difference (MCID) / Minimum important difference (MID) / Minimally Important Change (MIC) (MCIC) / Minimal clinically important improvement (MCII) / minimal relevant difference | 10 | 32.3% | 1.6% | 55.6% |
| Intraclass Correlation Coefficient ICC | 9 | 29.0% | 1.5% | 57.1% |
| Standard Error / Standard Error of Measure/ Standard Error of the Mean (SEM) / Standard Error of the Estimate | 9 | 29.0% | 1.5% | 58.6% |
| Interaction / Interaction effect / group-time interaction / group by session interaction / group-by-time interaction/time-by-group interaction | 9 | 29.0% | 1.5% | 60.0% |
| Mean change scores / mean change / mean difference | 8 | 25.8% | 1.3% | 61.3% |
| Interquartile/Interquartile range (IQR)/quartiles | 7 | 22.6% | 1.2% | 62.5% |
| Median | 7 | 22.6% | 1.2% | 63.7% |
| Independent / Dependent Variable(s) | 7 | 22.6% | 1.2% | 64.8% |
| Minimal Detectable Change (MDC) / Minimal Detectable Difference (MDD) / Least Detectable Difference (LDD) / Smallest detectable difference (SDDs) / smallest real difference (SRD) / relative value (SRD%) | 7 | 22.6% | 1.2% | 66.0% |
| A priori | 7 | 22.6% | 1.2% | 67.1% |
| Main Effects | 7 | 22.6% | 1.2% | 68.3% |
| Covariate / covariates / covariation | 7 | 22.6% | 1.2% | 69.4% |
| Continuous variables / continuous data | 7 | 22.6% | 1.2% | 70.6% |
| Sensitivity/Specificity | 6 | 19.4% | 1.0% | 71.5% |
| ANOVA (repeated measures): 2-way repeated ANOVA / 2x2 repeated-measures factorial ANOVA / mixed-effects, repeated measures ANOVA, 2 x 3 factorial repeated-measure ANOVA, lindear model for repeated measures | 6 | 19.4% | 1.0% | 72.5% |
| Descriptive Statistics/Variables (position and dispersion measurements)/Descriptive Analysis | 6 | 19.4% | 1.0% | 73.5% |
| Chi-Square test/chi square test / Hosmer-Lemeshow chi square / Pearson Chi-square / *X(2) / Pearson chi-square test of independence* | 6 | 19.4% | 1.0% | 74.5% |
| ANOVA (basic): 1-Way ANOVA /1-way repeated measures ANOVA / 2 by 2 ANOVA / 2 by 3 ANOVA / 2 by 3 factorial repeated-measure ANOVA /2 way ANOVA / 2 X 2 repeated-measures factorial ANOVA / 2 x 2 x 3 factorial ANOVA / 2 x 2 x6 ANOVA / 2-way multivariate ANOVA / 2-way random effects model ANOVA / 2-way repeated ANOVA / 3-way ANOVA / ANOVA for repeated measures / ANOVA F-type test / linear model for repeated / univariate 1 by 3 ANOVA measures ANOVA / 1-way random effects model / 2-way random effects model / random-effects variable / 2 x 2 x 4 linear mixed model / linear mixed model / 2-way mixed-effects model / 3 factor mixed-model analysis | 6 | 19.4% | 1.0% | 75.5% |
| non-parametric/ nonparametric / parametric | 6 | 19.4% | 1.0% | 76.5% |
| Categorical variables/ categoric variable | 6 | 19.4% | 1.0% | 77.5% |
| Sample size calculation/estimate | 5 | 16.1% | 0.8% | 78.3% |
| Pearson correlation coefficient r value/ r-value / Pearson product-moment correlation analysis | 5 | 16.1% | 0.8% | 79.1% |
| Cohen's d/d score/ cohen d/ d value | 5 | 16.1% | 0.8% | 79.9% |
| Type 1 error/Type 2 error/Type I/Type II | 4 | 12.9% | 0.7% | 80.6% |
| Linear Regression Analysis / multiple linear regression | 4 | 12.9% | 0.7% | 81.3% |
| Other Ratio | 4 | 12.9% | 0.7% | 81.9% |
| Wilcoxon signed rank test / Wilcoxon T / rank sum test / Wilcoxon matched pairs analysis / Wilcoxon Z | 4 | 12.9% | 0.7% | 82.6% |
| Pairwise Comparison /Pair-wise Correction/ Pairwise error rate (αPC) | 4 | 12.9% | 0.7% | 83.2% |
| Analysis of Covariance (ANCOVA) / 1-way ANCOVA /Univariate ANCOVA / 2-way ANCOVA | 3 | 9.7% | 0.5% | 83.7% |
| t value/t-value/t-score/t score/t statistic | 3 | 9.7% | 0.5% | 84.2% |
| Chronbach alpha / Cronbach alpha / Internal Consistency Coefficient | 3 | 9.7% | 0.5% | 84.7% |
| r^2^/R squared/Coefficient of determination | 3 | 9.7% | 0.5% | 85.2% |
| Variance Inflation Factor / variance | 3 | 9.7% | 0.5% | 85.7% |

Another 70 terms used 2 or fewer times.

# Table 6. Common Representative Statistical Terms: Cross Sectional Studies (n=29 articles)

|  | **Number of articles using term** | **Percentage of articles using term** | **Percent of all term occurrences** | **Cumulative Percentage** |
| --- | --- | --- | --- | --- |
| Minimum/Maximum/range | 27 | 93.1% | 4.7% | 4.7% |
| Standard Deviation / SD | 27 | 93.1% | 4.7% | 9.5% |
| P-Value/p value / p / probability value | 26 | 89.7% | 4.6% | 14.1% |
| Sample Size (N) | 25 | 86.2% | 4.4% | 18.5% |
| Percentage/Proportion/% | 25 | 86.2% | 4.4% | 22.8% |
| Mean/average | 24 | 82.8% | 4.2% | 27.1% |
| Statistical significance/difference/significant/significance/statistically significant | 21 | 72.4% | 3.7% | 30.8% |
| Intraclass Correlation Coefficient | 20 | 69.0% | 3.5% | 34.3% |
| Normally distributed/normal distribution / Normalized / Standardized / Normative Data/ Normality | 18 | 62.1% | 3.2% | 37.4% |
| F value/F-value / f statistic / F ratio | 16 | 55.2% | 2.8% | 40.2% |
| Pearson correlation coefficient r value/ r-value / Pearson product-moment correlation analysis | 14 | 48.3% | 2.5% | 42.7% |
| Descriptive Statistics/Variables (position and dispersion measurements)/Descriptive Analysis | 14 | 48.3% | 2.5% | 45.2% |
| Independent / Dependent Variable(s) | 12 | 41.4% | 2.1% | 47.3% |
| Significance level (α, alpha, corrected alpha value, level of significance) / alpha level / alpha coefficient / Criterion alpha (α) | 11 | 37.9% | 1.9% | 49.2% |
| Standard Error / Standard Error of Measure/ Standard Error of the Mean (SEM) / Standard Error of the Estimate | 10 | 34.5% | 1.8% | 51.0% |
| Between group differences/between-group/within-group differences/inter group/between-subjects/within-subject | 10 | 34.5% | 1.8% | 52.7% |
| Chi-Square test/chi square test / Hosmer-Lemeshow chi square / Pearson Chi-square / *X(2) / Pearson chi-square test of independence* | 9 | 31.0% | 1.6% | 54.3% |
| Median | 9 | 31.0% | 1.6% | 55.9% |
| post hoc analysis | 9 | 31.0% | 1.6% | 57.5% |
| Confidence Intervals (CI) | 8 | 27.6% | 1.4% | 58.9% |
| T-tests: 2-tailed p-values / 2-tailed t test / 2-sided t-test / 2-sided alternative hypothesis / 2-sample t-test /Independent t-test/independent sample t test / independent 1-tailed t-test / independent samples unequal variance t-test / unpaired t-test /Paired t-test/paired sample t-test/repeated groups' paired t test / related samples t-test / 1-sample t-test / student t-test / t statistic | 8 | 27.6% | 1.4% | 60.3% |
| Spearman Coefficients/spearman rank correlation (ρ)/ rho coefficient | 7 | 24.1% | 1.2% | 61.5% |
| Regression analysis / Regression model / Regression Lines / Regressor / Multiple Regression / Multivariable regression / Heirarchical multiple regression / Stepwise multiple regression (forward or backward) | 6 | 20.7% | 1.1% | 62.6% |
| Tukey | 6 | 20.7% | 1.1% | 63.6% |
| Wilcoxon signed rank test / Wilcoxon T / rank sum test / Wilcoxon matched pairs analysis / Wilcoxon Z | 6 | 20.7% | 1.1% | 64.7% |
| Power/Power Analysis | 5 | 17.2% | 0.9% | 65.6% |
| Unstandardized Coefficient (B/b) / Standardized Coefficient (Beta/β) | 5 | 17.2% | 0.9% | 66.4% |
| multiple or single-variable logistic regression analysis / multivariate logisitc regression analysis | 5 | 17.2% | 0.9% | 67.3% |
| ANOVA (basic): 1-Way ANOVA /1-way repeated measures ANOVA / 2 by 2 ANOVA / 2 by 3 ANOVA / 2 by 3 factorial repeated-measure ANOVA /2 way ANOVA / 2 X 2 repeated-measures factorial ANOVA / 2 x 2 x 3 factorial ANOVA / 2 x 2 x6 ANOVA / 2-way multivariate ANOVA / 2-way random effects model ANOVA / 2-way repeated ANOVA / 3-way ANOVA / ANOVA for repeated measures / ANOVA F-type test / linear model for repeated / univariate 1 by 3 ANOVA measures ANOVA / 1-way random effects model / 2-way random effects model / random-effects variable / 2 x 2 x 4 linear mixed model / linear mixed model / 2-way mixed-effects model / 3 factor mixed-model analysis | 5 | 17.2% | 0.9% | 68.2% |
| Other Ratio | 5 | 17.2% | 0.9% | 69.1% |
| Interaction / Interaction effect / group-time interaction / group by session interaction / group-by-time interaction/time-by-group interaction | 5 | 17.2% | 0.9% | 69.9% |
| Covariate / covariates / covariation | 5 | 17.2% | 0.9% | 70.8% |
| Multivariate analysis of variance (ANOVA) / Multiple analyses of variance (MANOVA) | 5 | 17.2% | 0.9% | 71.7% |
| Effect Size | 5 | 17.2% | 0.9% | 72.6% |
| Mann-Whitney U test | 5 | 17.2% | 0.9% | 73.5% |
| ROC curve | 5 | 17.2% | 0.9% | 74.3% |
| Mean change scores / mean change / mean difference | 4 | 13.8% | 0.7% | 75.0% |
| Wilk's lambda / wilks | 4 | 13.8% | 0.7% | 75.7% |
| Minimal Detectable Change (MDC) / Minimal Detectable Difference (MDD) / Least Detectable Difference (LDD) / Smallest detectable difference (SDDs) / smallest real difference (SRD) / relative value (SRD%) | 4 | 13.8% | 0.7% | 76.4% |
| Bonferroni Adjustment/Correction / Bonferroni-Dunn's procedure / bonferroni-holm test / bonferroni holm test | 4 | 13.8% | 0.7% | 77.2% |
| Confounding variable / confounders | 4 | 13.8% | 0.7% | 77.9% |
| Sensitivity/Specificity | 3 | 10.3% | 0.5% | 78.4% |
| A priori | 3 | 10.3% | 0.5% | 78.9% |
| Main Effects | 3 | 10.3% | 0.5% | 79.4% |
| Chronbach alpha / Cronbach alpha / Internal Consistency Coefficient | 3 | 10.3% | 0.5% | 80.0% |
| Pairwise Comparison /Pair-wise Correction/ Pairwise error rate (αPC) | 3 | 10.3% | 0.5% | 80.5% |
| Analysis of Covariance (ANCOVA) / 1-way ANCOVA /Univariate ANCOVA / 2-way ANCOVA | 3 | 10.3% | 0.5% | 81.0% |
| Likelihood ratios | 3 | 10.3% | 0.5% | 81.5% |
| Linear Regression Analysis / multiple linear regression | 3 | 10.3% | 0.5% | 82.1% |
| homoscedasticity / homogeneity of variance / homogeneity | 3 | 10.3% | 0.5% | 82.6% |
| Kappa | 3 | 10.3% | 0.5% | 83.1% |
| Area Under the Curve (AUC) | 3 | 10.3% | 0.5% | 83.7% |
| Floor / ceiling effect | 3 | 10.3% | 0.5% | 84.2% |
| Natural log transformations | 3 | 10.3% | 0.5% | 84.7% |
| parsimonious isometric multivariate model / parsimonious model | 3 | 10.3% | 0.5% | 85.2% |
| Clusters / cluster option | 3 | 10.3% | 0.5% | 85.8% |
| Collinearity / collinear | 3 | 10.3% | 0.5% | 86.3% |

Another 62 terms used 2 or fewer times.

# Table 7. Common Representative Statistical Terms: Systematic Reviews (n=19 articles)

| **Systematic Review** | **Number of articles using term** | **Percentage of articles using term** | **Percent of all term occurrences** | **Cumulative Percentage** |
| --- | --- | --- | --- | --- |
| Percentage/Proportion/% | 18 | 94.7% | 7.8% | 7.8% |
| Minimum/Maximum/range | 16 | 84.2% | 6.9% | 14.7% |
| Sample Size (N) | 15 | 78.9% | 6.5% | 21.2% |
| Mean/average | 13 | 68.4% | 5.6% | 26.8% |
| Standard Deviation / SD | 12 | 63.2% | 5.2% | 32.0% |
| P-Value/p value / p / probability value | 8 | 42.1% | 3.5% | 35.5% |
| Confidence Intervals (CI) | 8 | 42.1% | 3.5% | 39.0% |
| Statistical significance /difference/significant/significance/statistically significant | 5 | 26.3% | 2.2% | 41.1% |
| Intraclass Correlation Coefficient ICC | 5 | 26.3% | 2.2% | 43.3% |
| Sensitivity/Specificity | 5 | 26.3% | 2.2% | 45.5% |
| Effect Size | 5 | 26.3% | 2.2% | 47.6% |
| Odds Ratio / adjusted odds ratio | 5 | 26.3% | 2.2% | 49.8% |
| Pool/pooling/statistically pooled | 5 | 26.3% | 2.2% | 51.9% |
| Pearson correlation coefficient r value/ r-value / Pearson product-moment correlation analysis | 4 | 21.1% | 1.7% | 53.7% |
| Standard Error / Standard Error of Measure/ Standard Error of the Mean (SEM) / Standard Error of the Estimate | 4 | 21.1% | 1.7% | 55.4% |
| Median | 4 | 21.1% | 1.7% | 57.1% |
| Between group differences/between-group/within-group differences/inter group/between-subjects/within-subject | 4 | 21.1% | 1.7% | 58.9% |
| Chi-Square test/chi square test / Hosmer-Lemeshow chi square / Pearson Chi-square / *X(2) / Pearson chi-square test of independence* | 4 | 21.1% | 1.7% | 60.6% |
| Minimal Detectable Change (MDC) / Minimal Detectable Difference (MDD) / Least Detectable Difference (LDD) / Smallest detectable difference (SDDs) / smallest real difference (SRD) / relative value (SRD%) | 4 | 21.1% | 1.7% | 62.3% |
| Kappa | 4 | 21.1% | 1.7% | 64.1% |
| Meta-analysis | 4 | 21.1% | 1.7% | 65.8% |
| Relative Risk / risk ratio / adjusted risk ratio | 4 | 21.1% | 1.7% | 67.5% |
| Forest plot | 4 | 21.1% | 1.7% | 69.3% |
| Mean change scores / mean change / mean difference | 3 | 15.8% | 1.3% | 70.6% |
| Minimal Clinically important Difference (MCID) / Minimum important difference (MID) / Minimally Important Change (MIC) (MCIC) / Minimal clinically important improvement (MCII) / minimal relevant difference | 3 | 15.8% | 1.3% | 71.9% |
| Degree / Degrees of freedom / df | 3 | 15.8% | 1.3% | 73.2% |
| T-tests: 2-tailed p-values / 2-tailed t test / 2-sided t-test / 2-sided alternative hypothesis / 2-sample t-test /Independent t-test/independent sample t test / independent 1-tailed t-test / independent samples unequal variance t-test / unpaired t-test /Paired t-test/paired sample t-test/repeated groups' paired t test / related samples t-test / 1-sample t-test / student t-test / t statistic | 2 | 10.5% | 0.9% | 74.0% |
| Power/Power Analysis | 2 | 10.5% | 0.9% | 74.9% |
| Mann-Whitney U test | 2 | 10.5% | 0.9% | 75.8% |
| Variance Inflation Factor / variance | 2 | 10.5% | 0.9% | 76.6% |
| Area Under the Curve (AUC) | 2 | 10.5% | 0.9% | 77.5% |
| ROC curve | 2 | 10.5% | 0.9% | 78.4% |
| Percent of change / percent change / percent difference | 2 | 10.5% | 0.9% | 79.2% |
| Association | 2 | 10.5% | 0.9% | 80.1% |
| Median Change Scores / Median Difference between groups | 2 | 10.5% | 0.9% | 81.0% |
| Cohen interrater kappa value | 2 | 10.5% | 0.9% | 81.8% |
| homogenous / heterogenous / heterogeneity | 2 | 10.5% | 0.9% | 82.7% |
| weighted mean difference (WMD) / weighted standardized mean difference (WSMD) | 2 | 10.5% | 0.9% | 83.5% |
| I squared statistic / I squared | 2 | 10.5% | 0.9% | 84.4% |
| standardized mean difference (SMD) | 2 | 10.5% | 0.9% | 85.3% |

Another 34 terms used just once.
