# Supplementary material for: A bibliometric analysis of statistical terms used in American Physical Therapy Association journals (2011-2012): evidence for educating physical therapists
Source: BMC Med Educ. 2016 Apr 22;16:118. doi: 10.1186/s12909-016-0641-1 (PMC4840969; doi:10.1186/s12909-016-0641-1)
Supplement: Additional file 2: — Study design count and frequency for each journal. (DOCX 21 kb) [file 12909_2016_641_MOESM2_ESM.docx]

## Additional File 2 – Study Design by Journal

**Table 1 Study design count for each journal**

| **Study Design** | CPTJ | IJSPT | JACPT | JGPT | JNPT | JOSPT | JOPTE | JOWHPT | OPTP | PedPT | PTJ | PAL | RO | SH | Total |
| --- | --- | --- | --- | --- | --- | --- | --- | --- | --- | --- | --- | --- | --- | --- | --- |
| Prospective cohort | 4 | 23 | 1 | 8 | 10 | 19 | 3 | 4 | 1 | 19 | 31 | 0 | 1 | 3 | 127 |
| Case report | 2 | 8 | 4 | 2 | 0 | 5 | 4 | 3 | 14 | 6 | 15 | 0 | 3 | 0 | 66 |
| Randomized controlled trial | 1 | 2 | 0 | 1 | 1 | 7 | 1 | 1 | 0 | 0 | 17 | 0 | 0 | 0 | 31 |
| Cross sectional | 0 | 1 | 0 | 3 | 0 | 6 | 1 | 0 | 0 | 1 | 17 | 0 | 0 | 0 | 29 |
| Systematic review | 3 | 4 | 0 | 0 | 0 | 4 | 1 | 1 | 2 | 1 | 3 | 0 | 0 | 0 | 19 |
| Case series | 1 | 1 | 0 | 1 | 5 | 3 | 0 | 0 | 1 | 0 | 1 | 0 | 1 | 3 | 17 |
| Psychometric analysis | 0 | 1 | 1 | 0 | 1 | 6 | 0 | 0 | 0 | 2 | 5 | 1 | 0 | 0 | 17 |
| Retrospective cohort | 1 | 0 | 2 | 2 | 0 | 3 | 1 | 0 | 0 | 4 | 2 | 1 | 1 | 0 | 17 |
| Survey | 0 | 0 | 1 | 0 | 0 | 0 | 4 | 0 | 0 | 1 | 3 | 2 | 2 | 0 | 13 |
| Qualitative | 0 | 0 | 0 | 0 | 0 | 0 | 3 | 0 | 0 | 1 | 6 | 0 | 0 | 0 | 10 |
| Case control | 0 | 1 | 0 | 0 | 0 | 4 | 0 | 1 | 0 | 0 | 1 | 0 | 1 | 0 | 8 |
| Technological report | 0 | 1 | 0 | 0 | 1 | 1 | 0 | 0 | 0 | 1 | 2 | 0 | 0 | 0 | 6 |
| Secondary analysis | 0 | 0 | 0 | 1 | 0 | 1 | 0 | 0 | 0 | 0 | 4 | 0 | 0 | 0 | 6 |
| **Study Design** | CPTJ | IJSPT | JACPT | JGPT | JNPT | JOSPT | JOPTE | JOWHPT | OPTP | PedPT | PTJ | PAL | RO | SH | Total |
| Cross over | 2 | 1 | 0 | 0 | 0 | 2 | 0 | 0 | 0 | 1 | 0 | 0 | 0 | 0 | 6 |
| Cadaver or animal | 0 | 1 | 0 | 0 | 0 | 3 | 0 | 0 | 0 | 0 | 1 | 0 | 0 | 0 | 5 |
| Quasi experimental | 0 | 0 | 0 | 2 | 1 | 0 | 0 | 0 | 0 | 0 | 1 | 0 | 0 | 0 | 4 |
| Model analysis | 0 | 0 | 0 | 0 | 0 | 0 | 4 | 0 | 0 | 0 | 0 | 0 | 0 | 0 | 4 |
| Mixed method | 0 | 0 | 0 | 0 | 0 | 0 | 0 | 0 | 0 | 0 | 2 | 0 | 0 | 0 | 2 |
| Bibliometric analysis | 0 | 0 | 0 | 0 | 0 | 1 | 0 | 0 | 0 | 0 | 1 | 0 | 0 | 0 | 2 |
| Policy analysis | 0 | 0 | 0 | 0 | 0 | 0 | 0 | 0 | 0 | 0 | 1 | 0 | 0 | 0 | 1 |
| Economic analysis | 0 | 0 | 0 | 0 | 0 | 0 | 0 | 0 | 0 | 0 | 1 | 0 | 0 | 0 | 1 |
| **TOTAL** | **14** | **44** | **9** | **20** | **19** | **65** | **22** | **10** | **18** | **37** | **114** | **4** | **9** | **6** | 391 |

CPTJ = Cardiopulmonary Physical Therapy Journal; IJSPT = International Journal of Sports Physical Therapy; JACPT = Journal of Acute Care Physical Therapy; JGPT = Journal of Geriatric Physical Therapy; JNPT = Journal of Neurologic Physical Therapy; JOPTE = Journal of Physical Therapy Education; JOSPT = Journal of Orthoepaedic & Sports Physical Therapy; JWHPT = Journal of Women's Health Physical Therapy; OPTP = Orthopaedic Physical Therapy Practice; PAL = Physical Therapy Journal of Policy, Administration and Leadership; Ped PT = Pediatric Physical Therapy; PTJ = Physical Therapy Journal; RO = Rehabilitation Oncology; SH = Sports Health (“Sports Physical Therapy” articles)

**Table 2 Percentage of articles by study type for each journal**

| **Study Design** | **CPTJ** | **IJSPT** | **JACPT** | **JGPT** | **JNPT** | **JOSPT** | **JOPTE** | **JOWHPT** | **OPTP** | **PedPT** | **PTJ** | **PAL** | **RO** | **SH** |
| --- | --- | --- | --- | --- | --- | --- | --- | --- | --- | --- | --- | --- | --- | --- |
| Prospective cohort | 29% | 52% | 11% | 40% | 53% | 29% | 14% | 40% | 6% | 51% | 27% | 0% | 11% | 50% |
| Case report | 14% | 18% | 44% | 10% | 0% | 8% | 18% | 30% | 78% | 16% | 13% | 0% | 33% | 0% |
| Randomized controlled trial | 7% | 5% | 0% | 5% | 5% | 11% | 5% | 10% | 0% | 0% | 15% | 0% | 0% | 0% |
| Cross sectional | 0% | 2% | 0% | 15% | 0% | 9% | 5% | 0% | 0% | 3% | 15% | 0% | 0% | 0% |
| Systematic review | 21% | 9% | 0% | 0% | 0% | 6% | 5% | 10% | 11% | 3% | 3% | 0% | 0% | 0% |
| Case series | 7% | 2% | 0% | 5% | 26% | 5% | 0% | 0% | 6% | 0% | 1% | 0% | 11% | 50% |
| Psychometric analysis | 0% | 2% | 11% | 0% | 5% | 9% | 0% | 0% | 0% | 5% | 4% | 25% | 0% | 0% |
| Retrospective cohort | 7% | 0% | 22% | 10% | 0% | 5% | 5% | 0% | 0% | 11% | 2% | 25% | 11% | 0% |
| Survey | 0% | 0% | 11% | 0% | 0% | 0% | 18% | 0% | 0% | 3% | 3% | 50% | 22% | 0% |
| Qualitative | 0% | 0% | 0% | 0% | 0% | 0% | 14% | 0% | 0% | 3% | 5% | 0% | 0% | 0% |
| Case control | 0% | 2% | 0% | 0% | 0% | 6% | 0% | 10% | 0% | 0% | 1% | 0% | 11% | 0% |
| Technological report | 0% | 2% | 0% | 0% | 5% | 2% | 0% | 0% | 0% | 3% | 2% | 0% | 0% | 0% |

| **Study Design** | **CPTJ** | **IJSPT** | **JACPT** | **JGPT** | **JNPT** | **JOSPT** | **JOPTE** | **JOWHPT** | **OPTP** | **PedPT** | **PTJ** | **PAL** | **RO** | **SH** |
| --- | --- | --- | --- | --- | --- | --- | --- | --- | --- | --- | --- | --- | --- | --- |
| Secondary analysis | 0% | 0% | 0% | 5% | 0% | 2% | 0% | 0% | 0% | 0% | 4% | 0% | 0% | 0% |
| Cross over | 14% | 2% | 0% | 0% | 0% | 3% | 0% | 0% | 0% | 3% | 0% | 0% | 0% | 0% |
| Cadaver or animal | 0% | 2% | 0% | 0% | 0% | 5% | 0% | 0% | 0% | 0% | 1% | 0% | 0% | 0% |
| Quasi experimental | 0% | 0% | 0% | 10% | 5% | 0% | 0% | 0% | 0% | 0% | 1% | 0% | 0% | 0% |
| Model analysis | 0% | 0% | 0% | 0% | 0% | 0% | 18% | 0% | 0% | 0% | 0% | 0% | 0% | 0% |
| Mixed method | 0% | 0% | 0% | 0% | 0% | 0% | 0% | 0% | 0% | 0% | 2% | 0% | 0% | 0% |
| Bibliometric analysis | 0% | 0% | 0% | 0% | 0% | 2% | 0% | 0% | 0% | 0% | 1% | 0% | 0% | 0% |
| Policy analysis | 0% | 0% | 0% | 0% | 0% | 0% | 0% | 0% | 0% | 0% | 1% | 0% | 0% | 0% |
| Economic analysis | 0% | 0% | 0% | 0% | 0% | 0% | 0% | 0% | 0% | 0% | 1% | 0% | 0% | 0% |
| TOTAL | 100% | 100% | 100% | 100% | 100% | 100% | 100% | 100% | 100% | 100% | 100% | 100% | 100% | 100% |

CPTJ = Cardiopulmonary Physical Therapy Journal; IJSPT = International Journal of Sports Physical Therapy; JACPT = Journal of Acute Care Physical Therapy; JGPT = Journal of Geriatric Physical Therapy; JNPT = Journal of Neurologic Physical Therapy; JOPTE = Journal of Physical Therapy Education; JOSPT = Journal of Orthoepaedic & Sports Physical Therapy; JWHPT = Journal of Women's Health Physical Therapy; OPTP = Orthopaedic Physical Therapy Practice; PAL = Physical Therapy Journal of Policy, Administration and Leadership; Ped PT = Pediatric Physical Therapy; PTJ = Physical Therapy Journal; RO = Rehabilitation Oncology; SH = Sports Health (“Sports Physical Therapy” articles)
